# Customising Sustainable Bio-Based Polyelectrolytes: Introduction of Charged and Hydrophobic Groups in Cellulose

**DOI:** 10.3390/polym16223105

**Published:** 2024-11-05

**Authors:** Solange Magalhães, María José Aliaño-González, Pedro F. Cruz, Rose Rosenberg, Dirk Haffke, Magnus Norgren, Luís Alves, Bruno Medronho, Maria da Graça Rasteiro

**Affiliations:** 1 University of Coimbra, CERES, Department of Chemical Engineering, Pólo II–R. Silvio Lima, 3030-790 Coimbra, Portugal; solangemagalhaes@eq.uc.pt (S.M.); mgr@eq.uc.pt (M.d.G.R.); 2 Department of Analytical Chemistry, Faculty of Sciences, University of Cadiz, Agrifood Campus of International Excellence (ceiA3), IVAGRO, 11510 Puerto Real, Cadiz, Spain; mariajose.aliano@gm.uca.es; 3 MED–Mediterranean Institute for Agriculture, Environment and Development, Faculty of Science and Technology, University of Algarve, Campus de Gambelas, Ed. 8, 8005-139 Faro, Portugal; bfmedronho@ualg.pt; 4 CQC-IMS, Department of Chemistry, University of Coimbra, Rua Larga, 3004-535 Coimbra, Portugal; pjfc7@ci.uc.pt; 5 Physical Chemistry, Department of Chemistry, University of Konstanz, 78457 Konstanz, Germany; rose.rosenberg@uni-konstanz.de (R.R.); dirk.haffke@uni-konstanz.de (D.H.); 6 FSCN Research Centre, Surface and Colloid Engineering, Mid Sweden University, SE-851 70 Sundsvall, Sweden; magnus.norgren@miun.se

**Keywords:** cellulose, functionalisation, cationization, hydrophobicity, vegetable fatty acids

## Abstract

Cellulose has been widely explored as a sustainable alternative to synthetic polymers in industrial applications, thanks to its advantageous properties. The introduction of chemical modifications on cellulose structure, focusing on cationic and hydrophobic modifications, can enhance its functionality and expand the range of applications. In the present work, cationization was carried out through a two-step process involving sodium periodate oxidation followed by a reaction with the Girard T reagent, yielding a degree of substitution for cationic groups (DS_cationic_) between 0.3 and 1.8. Hydrophobic modification was achieved via esterification with fatty acids derived from commercial plant oils, using an enzyme-assisted, environmentally friendly method. Lipase-catalysed hydrolysis, optimised at 0.25% enzyme concentration and with a 1 h reaction time, produced an 84% yield of fatty acids, confirmed by FTIR and NMR analyses. The degree of substitution for hydrophobic groups (DS_hydrophobic_) ranged from 0.09 to 0.66. The molecular weight (MW) of the modified cellulose derivatives varied from 1.8 to 141 kDa. This dual modification strategy enables the creation of cellulose-based polymers with controlled electrostatic and hydrophobic characteristics, customisable for specific industrial applications. Our approach presents a sustainable and flexible solution for developing cellulose derivatives tailored to diverse industrial needs.

## 1. Introduction

Sustainable development aims to mitigate the adverse environmental impacts associated with economic growth and globalisation. It seeks solutions to the challenges posed by industrialisation and raw-material depletion. This concept encompasses more than just ecological improvements; it includes prudent resource utilisation and efficient energy use, providing a holistic approach to tackling the interconnected and demanding challenges of our time.

One important strategy to reduce environmental impact is the development and use of biobased materials, which have received substantial research interest. Natural polymers derived from biomass resources, such as cellulose, are expected to play a crucial role in future materials. Cellulose, an abundantly available polysaccharide, can be sourced from wood, forestry residues, agricultural waste, and grasses [1,2]. Its versatility spans diverse applications, including cellulose derivatives used in food, cosmetics, and pharmaceuticals, as well as cellulose nanomaterials [3,4,5,6]. Additionally, cellulose is essential in producing pulp and paper, packaging, and other biopolymers [7,8].

As explored in several studies, cellulose has emerged as a promising alternative to synthetic polymers in various industrial applications [2,9]. Despite its advantageous properties, cellulose faces limitations, such as poor solubility in common solvents and lack of thermoplasticity [10]. To overcome these constraints, cellulose can undergo controlled chemical modifications, often preceded by pretreatments to enhance its reactivity and versatility. For instance, Hashem et al. [11] investigated the chemical modification of cotton fabric via cationization with 3-chloro-2-hydroxypropyltrimethylammonium chloride in a strongly alkaline medium. This process forms cationized cellulose through an ether linkage but also suffers from a competing hydrolysis reaction, compromising reaction efficiency. Aulin et al. have also evaluated the synthesis of cationic cellulose micro/nanofibres using similar reagents [12]. Another approach, explored by Grenda et al. [13], involved cellulose oxidation with sodium periodate, followed by reactions with cationic or/and anionic agents. This process introduces highly reactive aldehyde groups in the cellulose chain, forming dialdehyde cellulose (DAC), which can be further modified to introduce cationic or anionic moieties, enhancing cellulose solubility and functionality. Similar pathways have been explored in studies using various pulp sources [14,15,16].

Another possible functionalisation of cellulose involves the introduction of hydrophobic modifications. In this regard, Beaumont et al. introduced aliphatic esters onto the cellulose surface via esterification [17]. Alternatively, Wei et al. suggested the synthesis of hydrophobic cellulose by dissolving wood pulp with canola oil fatty acid methyl ester, leading to a transesterification reaction [18]. The interested reader is referred to recent reviews that also focus on cellulose modification [2,9,19].

The present work seeks to study a two-step modification of cellulose derived from Acacia wood, an invasive species in Portugal, involving the formation of cationic dialdehyde cellulose (CDAC) followed by the synthesis of hydrophobic cationic dialdehyde cellulose (HCDAC). This dual modification creates a functional biopolymer, capable of establishing ionic and hydrophobic interactions and therefore with potential application in areas such as flocculation, detergency, and cosmetics. A complete library of CDAC and HCDAC was developed with tunned charge density and hydrophobicity degree. The novel cellulose-based derivatives were extensively characterised by different methods, such as FTIR-ATR, NMR, surface tension, and charge density, among others.

## 2. Materials and Methods

### 2.1. Materials

The cellulose used in this study was extracted from *Acacia dealbata* wood collected in Midões (Tábua, Portugal). The raw material was finely ground in a laboratory mill (Cross Beater Mill, Retsch, Haan, Germany) and screened in a mechanical sieve shaker (Thomas Scientific, Swedesboro, NJ, USA) to obtain a sample with a particle size between 0.25 and 0.84 µm, which was selected for the subsequent pretreatment. The sample was fractionated using a DES mixture composed of choline chloride (99%, ChCl) and lactic acid (98%, LA), which were obtained from Acros Organics (Thermo Fisher Scientific, Waltham, MA, USA) following the procedure previously described by us [20]. In brief, the DES (ChCl:LA 1:2) was mixed with the wood powder, using a ratio of wood/solvent of 1.5:10 (biomass dry weight to solvent weight) and heated in a metal reactor for 4 h at 160 °C. After the extraction, the resulting solid residue (cellulose-rich fraction) was separated from the DES by vacuum filtration. A 10 wt% NaOH (aq) was used to wash the cellulose-rich fraction. The obtained cellulose fraction was then oven-dried and weighed. Table 1 presents the main components of the native Acacia wood and the extracted cellulose fraction.

The lignin content of the extracted cellulose-rich fraction was estimated using the National Renewable Energy Laboratory (NREL) protocol [21]. In parallel, the composition of the samples in terms of cellulose and hemicellulose was determined by high-performance liquid chromatography (HPLC), following the protocol described in Section 2.5 (NREL protocol). The cellulose-rich fractions were hydrolysed following the same procedure used for lignin determination, and then characterised by HPLC system (KNAUER AZURA^®^ liquid chromatography instruments, Berlin, Germany) equipped with a refractive index (RI) detector. A Rezex (Alcobendas, Spain) ROA-Organic Acid H+ column (300 × 7.8 mm) maintained at 40 °C, along with a guard column at room temperature, was employed. Sample injections of 20 μL and a mobile phase of 0.0025N H_2_SO_4_ aqueous solution, pre-filtered with a 0.2 μm nylon membrane (Whatman, Buckinghamshire, United Kingdom), at a flow rate of 0.6 mL·min^−1^, were used for all HPLC assays to quantify cellulose content [22].

The following chemicals were sourced from Sigma-Aldrich (Algés, Portugal) at analytical grade and used for the synthesis and characterisation of cellulose derivatives: sodium periodate (NaIO_4_), hydroxylamine hydrochloride (NH_2_OH·HCl), acetic acid (CH_3_COOH), and sodium acetate trihydrate (CH_3_COONa·3H_2_O). For the preparation of water-soluble HCDACs, 2-hydrazinyl-2-oxoethyl)-trimethylazanium chloride (Girard’s reagent T or simply abbreviated as GT), potassium carbonate (K_2_CO_3_), methanol, hexane, and diethyl ether were purchased from Sigma-Aldrich (Algés, Portugal) with p.a. grade and used without further purification. Hydrochloric acid (37%) and 2-propanol were supplied by the VWR company (Carnaxide, Portugal).

The acetate buffer solution (pH 4.5) used in the oxime reaction was prepared by dissolving 27.4 g of sodium acetate trihydrate (≥99.0%, Sigma-Aldrich, Algés, Portugal) and 15 mL of glacial acetic acid (99%, Fisher Chemical, Porto Salvo, Portugal) in a 2 L volumetric flask. The mixture was then diluted to a final volume of 2 L with deionized water.

For the extraction of fatty acids, a commercial vegetable oil, sourced from a local supermarket, was used. The vegetable oil contained a total fat content of 100 g, comprising 8.0 g of saturated, 63 g of monounsaturated, and 29 g of polyunsaturated lipids. The lipase LJP30162 was obtained from Aquitex (Pedrouços, Portugal).

For NMR, the deuterated chloroform (CDCl_3_), the sodium 3-(trimethylsilyl) propionate-d4, the deuterium oxide, and the NMR tubes were purchased from Sigma-Aldrich (Algés, Portugal).

### 2.2. Extraction of Fatty Acids

Fatty acids were extracted from a commercial vegetable oil using the enzymatic hydrolysis approach developed by Magalhães et al. with some adjustments [21]. The enzymatic hydrolysis was performed at room temperature and atmospheric pressure, using water as solvent. In brief, the reaction was conducted in a glass beaker containing 0.5 g (0.25% (*w*/*v*)) to 1 g (0.5% (*w*/*v*)) of lipase and 200 mL of a solution of water and the commercial vegetable oil (50:50, *w*/*w*), for 1 h and stirred at 500 rpm. After completing the reaction, dietylether was added to separate the sample into 3 phases. The fatty acids-rich fraction (solid phase) was dried in an oven to remove any remaining solvent.

### 2.3. Cationization of Cellulose

**Figure 1:**
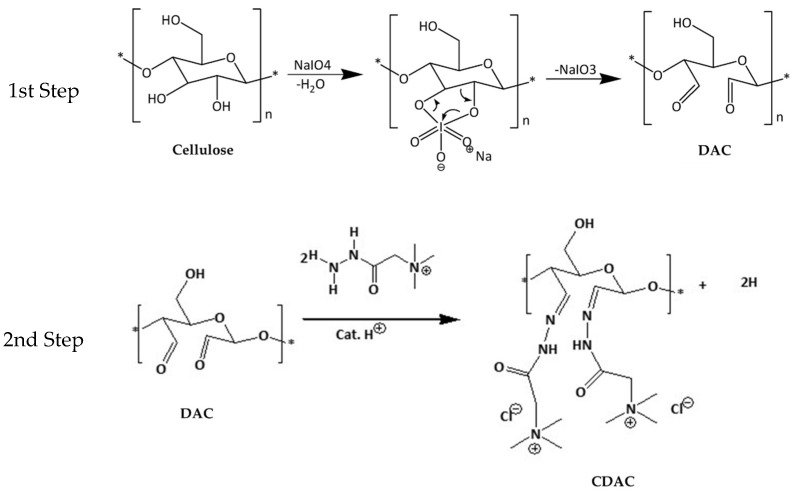
Schematic representation of the 2-step reactions involved in the preparation of cationic cellulose (CDAC).

The cationic functionalisation of the cellulosic materials was achieved through a dual-step cationization process (Figure 1), which involved the initial oxidation of cellulose to dialdehyde cellulose (DAC) and subsequent cationization with the GT reagent.

The procedure follows a methodology previously developed by Kinga et al. [23]. Briefly, 4 g (dry basis) of cellulose was dispersed in 2 L of distilled water (4% consistency) and stirred overnight with a magnetic stirrer to fully hydrate the cellulose. The resulting suspension was then transferred to a round-bottom reaction flask, where precise amounts of NaIO_4_ (7.2 g) and LiCl (8.2 g) were added. The mixture was then diluted with 200 mL of distilled water. The reaction vessel was covered with aluminium foil to prevent the photo-induced decomposition of periodate and placed in an oil bath. According to Sirviö et al. [24], LiCl can act as a catalyst, thereby enhancing the oxidation efficiency. The improvement obtained can be attributed to the ability of the lithium ions to disrupt the hydrogen bonds between the cellulose chains, thereby facilitating the interaction between the chemical reagents and the cellulose chains. After the reaction was completed, the product was filtered and washed several times with distilled water to remove any residual iodine compounds from the obtained DAC. The aldehyde content of the oxidised cellulose was determined by reacting the DAC (ca. 0.1 g) with 1.39 g of NH_2_OH·HCl, dissolved in 100 mL of 0.1 M acetate buffer (pH = 4.5). Subsequently, the mixture was subjected to stirring and left to react for 48 h. Finally, the product was filtered, washed with distilled water, dried, and subjected to elemental analysis EA 1108 CHNS-O from Fisons. A tin capsule with a certain amount of sample (2–3 mg) was introduced into a vertical quartz tube reactor heated at 900 °C with a constant He stream. Before dropping the sample into the combustion tube, the He stream was enriched with oxygen to achieve a strong oxidising environment which guarantees complete combustion/oxidation. The resulting four components of the combustion mixture were eluted into a chromatographic column and then detected by a thermal conductivity detector, in the sequence N_2_, CO_2_, H_2_O, and SO_2_. The 2,5-Bis(5-tert-butyl-benzoxazol-2-yl)thiophene, was used as standard. Note that 1 mol of aldehyde stoichiometrically reacts with 1 mol of NH2OH∙HCl, resulting in 1 mol of the oxime product, Therefore, the aldehyde content can be calculated directly from the nitrogen content of the product.

The cationization of DAC was achieved by redispersing it in distilled water (0.8 g dry basis in 80 mL of water) and adding the GT reagent. From the literature [14,23,25], the selected GT/aldehyde ratios were 7.8, 3.9, or 1.95. The pH of the mixture was adjusted to 4.5 with HCl (1M) and then left to react at 70 °C for 1 h. After the reaction was concluded, the obtained mixture was diluted with isopropanol to precipitate the insoluble products (i.e., cationic DAC). The precipitated material was centrifuged and washed with a water/isopropanol mixture (1:9, *v*/*v*) to remove any unreacted GT reagent.

### 2.4. Hydrophobic Modification

**Figure 2:**
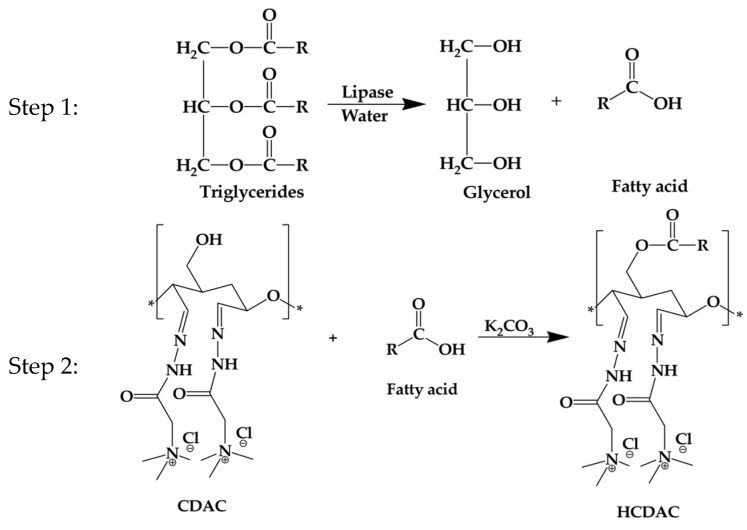
Schematic representation of the fatty acids’ extraction (step 1) and CDAC hydrophobisation (step 2).

The hydrophobic modification of CDAC was initiated by dissolving the catalyst K_2_CO_3_ (concentrations ranging from 0.11 to 0.18 g, in 10 mL MeOH), with constant stirring for 30 min. Subsequently, the CDAC and the fatty acids were added to the reaction media with a ratio of 1:1, 1:2, or 1:3 (*w*/*w*), and the reaction was left to continue for 3 to 24 h (t), at temperatures (T) ranging from 50 to 90 °C. Once the reactions were completed, the condenser was removed from the apparatus, and the mixture was left at 90 °C to remove MeOH. After the total MeOH evaporation, hexane was added to the obtained pellet, and the hydrophobically modified CDAC (HCDAC) was recovered by filtration. The addition of hexane enables the removal of unreacted fatty acids from the reaction product. The HCDAC was then dried under vacuum overnight. The suggested reaction mechanism is schematically depicted in Figure 2.

### 2.5. Characterisation of Extracted Fatty Acids

The extracted fatty acids were characterised using the Fourier transform infrared (FTIR) spectroscopic method with attenuated total reflectance (ATR) and proton nuclear magnetic resonance, ^1^H NMR. The FTIR-ATR spectra of the fatty acids were recorded in a JASCO FT/IR-4200 spectrometer (Jasco Corporation, Tokyo, Japan) using a universal ATR accessory, in the range of 500–4000 cm^−1^, with a resolution of 4 cm^−1^ and applying 128 scans. For the NMR assays, the extracted fatty acids were solubilised in deuterated chloroform 10% (*v*/*v*). The spectra were obtained using a Bruker Avance III 400 MHz spectrometer (Bruker, Hamburg, Germany), operating at frequencies of 400.13 MHz (^1^H). All the spectra were obtained at 25 °C using standard pulse sequence zg30, with a spectral window of 8417.5 Hz a total of 32 k points, acquisition time of 1.9 s with a relaxation delay (d1) of 1 s for a total of 32 scans. Sodium 3-(trimethylsilyl) propionate-d4 (TMSP) served as the internal standard for calibration.

### 2.6. Degree of Substitution of the CDAC

The nitrogen content resulting from of the oxime derivative in CDAC was estimated using an elemental analyser Fisons EA 1108 CHNS-O (Thermo Fisher, Waltham, MA, USA). A tin capsule with a weighted amount of sample was introduced into a vertical quartz tube reactor and heated at 900 °C with a constant helium stream. Before dropping the sample into the combustion tube, the helium stream was enriched with oxygen to achieve a strong oxidising environment, which guarantees complete combustion/oxidation. The resulting four components of the combustion mixture were eluted into a chromatographic column and then detected by a thermal conductivity detector, in the sequence N_2_, CO_2_, H_2_O, and SO_2_. BBOT-2,5-Bis(5-tert-butyl-benzoxazol-2-yl)thiophene was used as standard. From the elemental analysis, the nitrogen content was obtained in the form of a mass percentage (N%), which corresponds to the mass of nitrogen (mN) divided by the total mass of the sample (mT) (Equation (1)).

(1) $$N\%=\frac{m_{N}}{m_{T}}\times 100=\frac{DS\times 14}{{MW}_{AGU}+DS\times ({MW}_{sub}-{MW}_{lg})}\times 100$$

In this equation, 14 is the atomic mass of the nitrogen atom, MW_AGU_ is the molar mass of the AGU unit, MW_sub_ represents the molar mass of the substituent groups, and MW_lg_ is the molar mass of the group leaving the AGU during the reaction.

Note that the nitrogen content must be divided by 3, since each substituent group contains three nitrogen atoms. The molar fraction of cationic units and cationicity index can be estimated from the following equations:

(2) $$Amount\;of\;cationic\;units\left(Ac\right)=\frac{\frac{N\%}{100}\times 458.9}{14\times 6}[/g]$$

(3) $$molar\;fraction\;of\;cationic\;units=\frac{mol\;of\;cationic\;units}{total\;mol}=\frac{\frac{Ac}{458.9}}{\frac{Ac}{458.9}+\frac{1-Ac}{162}}$$

In the case of the cationization with GT, the DS will be multiplied by 2, since each AGU can contain two substituent groups.

(4) $$DS=\frac{mol\;of\;cationic\;units\times 2}{total\;mol}=\frac{2\times \frac{Ac}{458.9}}{\frac{Ac}{458.9}+\frac{1-Ac}{162}}$$

(5) $$ationicity\;index=\frac{Ac}{458.9}\times 2\times 1000[\frac{mmol}{g}]$$

where 162 g·mol^−1^ corresponds to the molar mass of the AGU unit and 458.9 g·mol^−1^ corresponds to the molar mass of the cationic unit. It is also assumed that, in the final product, only AGU and cationic units are present (no aldehyde groups are present).

### 2.7. Degree of Substitution of HCDAC

The prepared hydrophobically modified cationic cellulose derivatives were characterised using a set of techniques comprising FTIR-ATR, ^1^H NMR, elemental analysis, and analytical ultracentrifugation. FTIR, elemental analysis, and ^1^H NMR were performed applying the conditions and the instrument setups previously described in Section 2.5. For the NMR measurement, the HCDAC derivatives were dissolved in D_2_O (10 mg·mL^−1^) directly within the NMR tube to mitigate any potential contamination. The hydrophobic DS was determined by NMR, following the integrating of the peak at 0.82 ppm, which is characteristic of the CH_3_ groups in the fatty acids, and the peak at 4.07 ppm corresponding to carbon C6 in the cellulose backbone. The DS was estimated from the ratio of the two mentioned integrals (Equation (6)).

(6) $$DS=\frac{Area\;from\;peak\;at\;4.07\;\mathrm{ppm}}{Area\;from\;peak\;at\;0.82\;\mathrm{ppm}}\times 100$$

### 2.8. Molecular Weight of the Hydrophobically Modified Cationic Cellulose

The molecular weight of hydrophobically modified cationic cellulose was determined by using Analytical Ultracentrifugation (AUC). The setup consisted in a UV–vis multiwavelength detector [26,27,28] based on a modified Optima XL 80k ultracentrifuge (Beckman Coulter, CA, USA), Ti double sector cells (Nanolytics GmbH, Potsdam, Germany) with 1.2 cm centrepieces, and sapphire windows. The sedimentation velocity experiments were performed at 60,000 rpm and 20 °C, using 1 mg·mL^−1^ solutions of the cellulose derivatives dissolved in Milli-Q water. The SEDFIT version 16.36 was used for performing a high-resolution sedimentation coefficient distribution c(s) by the maximum entropy regularisation method and transforming it into a molar mass distribution c(M) [29] with a resolution of 100 grid points and a confidence level of 0.68. For these calculations, the density of the prepared derivatives, and the density and viscosity values of water (i.e., 0.01002 Pa·s, *ρ*_0_ = 0.99823 g·mL^−1^) at 20 °C were used. Density measurements were performed on a density meter DMA 5000 M (Anton Paar, Graz, Austria) at 20 °C. The density of hydrophobically modified cationic cellulose solute (*ρ*_i_) was determined by taking into account of the Kratky density balance using Equation (7) [30]. Figure S1 in the Supplementary Materials shows the measured densities (*ρ*) for the different prepared derivatives with different concentrations (c).

(7) $$\frac{1}{\rho_{i}}=\frac{1}{\rho_{0}}\left[1-\left(\frac{\rho -\rho_{0}}{c}\right)\right]$$

A detailed record of the calculated molar mass distributions c(M) for the different HCDAC polymers is shown in the Supplementary Figure S2.

### 2.9. Zeta Potential Determination

A Zetasizer NanoZS from Malvern Instruments (Malvern Instruments, Malvern, UK) was used to measure the zeta potential of the cationic cellulose polymers in solution. Each sample was measured 5 times with 15 sub-runs for each measurement. In short, a stock solution of modified cellulose was prepared (0.2 g of modified cellulose in 100 mL of water). The solutions were magnetically stirred for 1 h and then submitted to sonication for 5 min at 50 kHz. The zeta potential was measured at 25 °C with an equilibration time of 60 s.

### 2.10. Surface Tension Measurement

The surface tension between various HCDAC solutions and air was measured using the force tensiometer K20 (Krüss, Hamburg, Germany), equipped with a Wilhelmy plate. Prior to each measurement, thorough cleaning of the plate and beakers was conducted by sequentially flushing them with acetone, ethanol, and water, followed by drying and plasma cleaning for 3 min. This meticulous cleaning process was repeated before every measurement. Furthermore, plate calibration was performed by measuring the surface tension between air and water, typically yielding values in the range of ca. 72.5–73 mN·m^−1^. Measurements were conducted at regular intervals of 5 min over a period of 60 min for each sample to ensure accuracy and consistency.

### 2.11. Statistical Analysis

The Box–Behnken design is a type of response surface methodology (BBD-RSM) employed in experimental design to optimise complex processes by evaluating the effects of multiple variables and their interactions. Unlike other designs, Box–Behnken does not include combinations wherein all variables are at their extreme values, which enhances safety and efficiency, particularly for experiments with constraints. This design requires fewer runs than a full factorial design, making it cost-effective and timesaving. The structured, spherical arrangement of experimental points allows for more precise and reliable modelling of the response surface, facilitating the identification of optimal conditions and interactions among variables with greater accuracy. For this reason, in the current research, a BBD-RSM design was employed to evaluate the influence of the reaction conditions on the synthesis of hydrophobised cationic cellulose-based flocculants from Acacia wood. For that, three variables were selected. The ratio of CDAC to fatty acid (1:1–1:3), the time (3–24 h), and the temperature (50–90 °C) were the variables selected for this study. The K_2_CO_3_ concentration and the amount of methanol were fixed at 0.01 g·mL^−1^ and 10 mL, respectively.

In addition, a *t*-test with a confidence level of 95% was performed to calculate the *p*-values for each of the variables studied. The variables yielding *p*-values below 0.05 were considered to have a significant influence on the response. The ANOVA and the BBD-RSM design were generated and analysed using the statistical software Statgraphic Centurion (version XVII) (Statgraphics Technologies, Inc., The Plains, VA, USA).

## 3. Results and Discussion

### 3.1. Enzymatic Extraction of Fatty Acids

Lipases, a versatile family of enzymes, are particularly adequate for the hydrolysis of triglycerides into free fatty acids and glycerol. Their efficient catalyst performance is further evidenced by their capacity to operate under moderate temperatures and pressures. The initial experiments focus on determining the optimal concentration of lipase and reaction conditions to achieve efficient outcomes, as shown in Table 2. The water content was kept identical to that of the vegetable oil, as it plays a pivotal role in enzymatic transesterification.

From Table 2, it can be observed that, as the concentration of lipase increases, the fatty acid yield decreases. This phenomenon may be attributed to the effects of enzymatic agglomeration, previously described for other lipases in their free form [31]. Consequently, for subsequent experiments, the optimal amount of catalyst was established at 0.25% (*w*/*v*), to achieve a balance between enhanced enzymatic activity and mitigating the adverse effects associated with increased lipase quantity.

**Figure 3:**
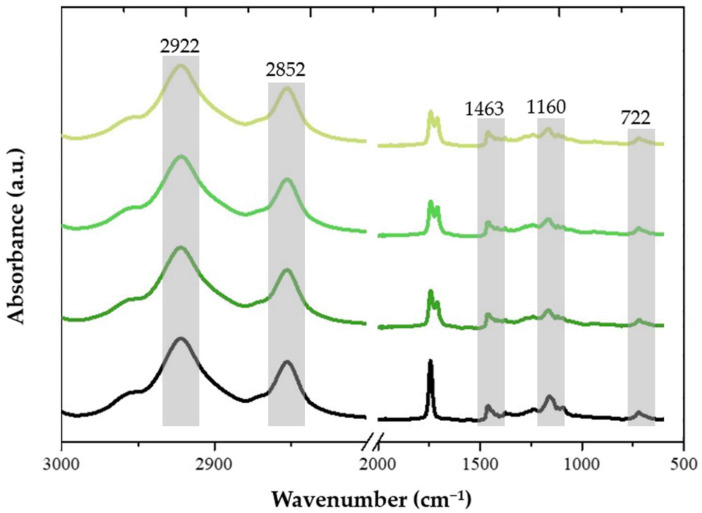
FTIR analysis of the isolated fatty acids obtained after 1 h (dark green), 2 h (light green), 3 h (yellow). The native vegetable oil (black spectrum) is presented for comparison.

Following the isolation of fatty acids, a comprehensive analysis and characterisation of each fraction was conducted. The qualitative analysis of different fatty acid samples was performed through FTIR analysis. Figure 3 presents a comparative display of the normalised spectra for the oil samples after different enzymatic treatment times.

The vibrational bands observed at 2922 and 2852 cm^−1^ are assigned to the stretching vibrations of C-H bonds in the CH_2_ aliphatic groups [32]. Notably, their intensity is consistently high across all oil samples, which is particularly characteristic of triglycerides. Another manifestation related to aliphatic groups is the band at 1463 cm^−1^, indicative of bending vibrations in both CH_2_ and CH_3_ groups, and this characteristic remains similar among the various samples. The C=O group manifests its presence with a brand centred at 1742 cm^−1^, and interestingly, this band is also evident across the different samples. The vibration mode associated with the C-O ester bonds of triglycerides is found at ca. 1160 cm^−1^ and remains rather unchanged between samples. Likewise, the band at 722 cm^−1^, corresponding to the overlap of CH_2_ vibrations and vibrations of cis-disubstituted olefins, remains consistent for all samples [33]. Overall, the identified vibrational modes in the fatty acid spectra align with the main characteristic bands reported in the literature. No significant differences are observed among the different samples of fatty acids. Since no remarkable differences in yield or main functional groups were found after 1h of enzymatic reaction, 1 h was considered suitable to extract the targeted fatty acids.

**Figure 4:**
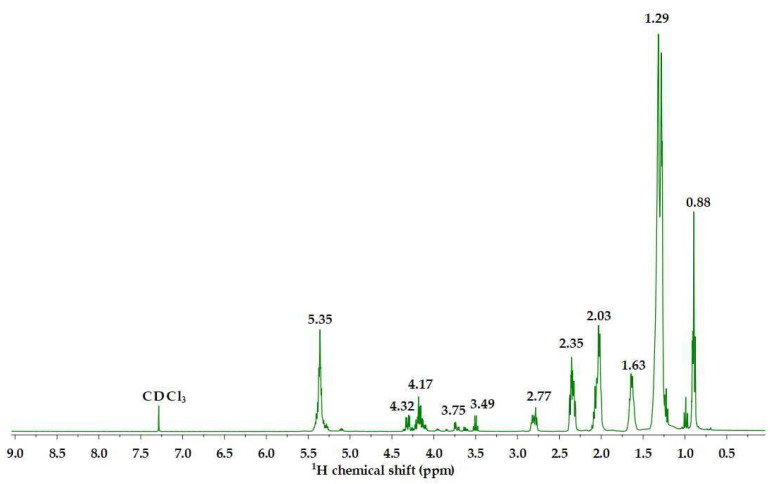
^1^H NMR spectrum of the fatty acids extracted from the commercial vegetable oil after 1 h enzymatic reaction and using 0.25% (*w*/*v*) of lipase.

The fatty acids extracted after 1 h of enzymatic reaction were further characterised by ^1^H NMR (Figure 4).

As can be observed in Figure 4, the NMR data evidence the exclusive presence of fatty acids in the samples. This is supported by the disappearance of the doublets within the 4.14–4.30 ppm range, associated with the glyceryl groups, and the multiplet observed at 5.25–5.30 ppm, which is assigned to the CH group in glycerol. The fact that these signals have vanished strongly implies the successful cleavage of the ester bonds of the triglycerides to form fatty acids. Furthermore, the appearance of the triplet signal, attributed to the α-methylene proton of the acid, was observed at 2.35 ppm. The -CH_2_ signals of these fatty acids appear at higher chemical shifts than their ester counterparts, owing to the heightened deshielding effect exerted by the carboxylic group in contrast to the ester group. The higher chemical shifts result from the greater susceptibility of protons within carboxylic groups to the magnetic field in NMR spectroscopy, relative to those in ester groups.

### 3.2. Cellulose Oxidation

Once the extraction of fatty acids was completed and cellulose obtained from the Acacia wood residues, the work progressed toward its functionalisation. The process was initiated with cellulose cationization which, as described in the experimental section, is composed of an initial oxidation step followed by the reaction with the GT reagent (see Figure 1 for details). The different reaction conditions used are detailed in Table 3.

The results displayed in Table 3 reveal that increasing the molar ratio of NaIO_4_ leads to a higher degree of dialdehyde substitution in the cellulose structure. This observation agrees with the findings of Pedrosa et al. and Grenda et al., who noticed a direct relationship between the amount of oxidizing agent and the DS of DAC [14,34].

The obtained DACs with different aldehyde contents were treated with the GT reagent following the conditions highlighted in Table 4 and the procedure described in Section 2.3.

The GT/aldehyde molar ratio played a crucial role in controlling the degree of cationization, offering a means to adjust the cationicity index of the resulting products. Notably, a high cationicity index could still be achieved even with a lower aldehyde content in the initial DAC by increasing the GT/aldehyde ratio. In general, a higher GT/aldehyde molar ratio was associated with greater cationicity, particularly when comparing results from the same starting material at a consistent temperature of 70 °C. These observations are consistent with previous studies, especially those by Grenda et al. [13,23,34]. However, the relationship between cationicity and zeta potential is not straightforward. As the cationic charge increases, the zeta potential decreases. In polymers with lower cationic DS, aggregation may occur, but for highly charged polymers, significant particle aggregation is not expected. While polymer aggregation can contribute to a reduction in zeta potential, it does not fully explain the observed trend. Other factors, such as the strength of interaction between charged groups and counterions, also affect zeta potential measurements. Polymers with higher-charge density likely exhibit stronger interactions with counterions, which may reduce the movement of surrounding water by increasing local viscosity. This effect leads to lower zeta potential values for polymers with high-charge density [35], a phenomenon documented in previous studies. These findings emphasise the importance of charge distribution in determining the stability and behaviour of cationic polymers in suspension [36].

**Figure 5:**
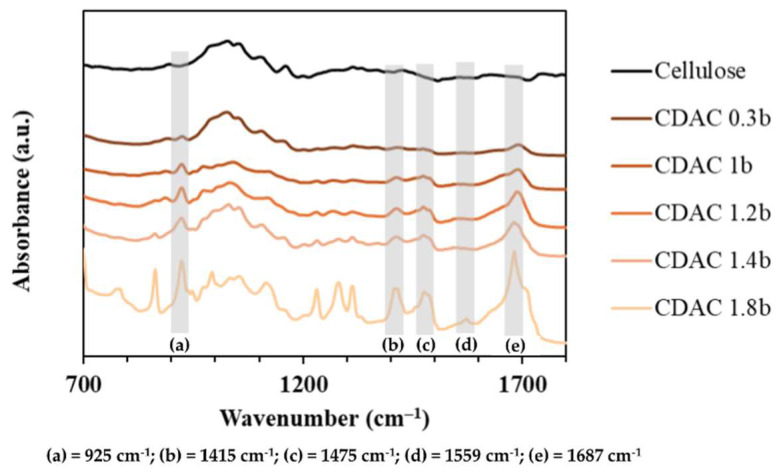
FTIR spectra of CDAC cellulosic samples with different DS (i.e., 0.3, 1, 1.2, 1.4, 1.8). The main vibrational modes are highlighted.

The FTIR analysis revealed a linear correlation between the DS and the intensity of specific bands resulting from the cationization reaction, as shown in Figure 5.

The cationization is evidenced by the appearance of a new peak at 1687 cm^−1^, corresponding to the stretching of the carbonyl group in the hydrazide structure of GT. The formation of an imine bond between the aldehyde groups of DAC and GT is further confirmed by a peak at 1559 cm^−1^. The bands at 1475 cm^−1^ and 1415 cm^−1^ can be attributed to the asymmetric and symmetric bending of the methyl groups in GT, respectively. Additionally, the peak at 925 cm^−1^ can be ascribed to the asymmetric stretching of the C-N bond in quaternary ammonium, the N-N bond of GT, or even the C-C stretching vibration of the GT linkage [14].

Hydrophobisation was conducted following cationization, to ensure that the esterification reaction with the fatty acids does not compromise the stability of the cationic substitution. The hydrophobic index of the modified cellulose was adjusted through experimental design by varying the reaction parameters, including temperature, reaction time, and different weight ratios of fatty acids to cationic cellulose groups, as detailed in Table 5.

The hydrophilisation started with the addition of the chosen cationic derivative (CDAC 1.4b), selected for its intermediate DS, to the fatty acid extracted from vegetable oil and the catalyst (K_2_CO_3_) in methanol to allow for the esterification reaction (see experimental Section 2.4 for details). As can be observed in Table 5, temperature stands out as the parameter that most affects the HCDAC yield. This observation aligns with findings from various previous studies, which indicate that elevated temperatures improve the efficiency of esterification reactions by promoting better molecular interactions and catalyst activity [18]. Note that the catalyst concentration was not increased, as the objective was to prevent the formation of a cellulose derivative with excessively high hydrophobicity, in order to ensure its solubility in water.

As described in Section 2.11, a BBD-RSM design was employed to evaluate the influence of reaction conditions on the synthesis of the novel HCDACs. Three key variables were selected: the ratio of CDAC to fatty acid (1:01–03), reaction time (3–24 h), and temperature (50–90 °C). The BBD-RSM data presented in Table 6 were subjected to an ANOVA test at the 95% confidence level, with yield as the response variable. The developed analytical method yielded an R^2^ of 80.80%, while the Durbin–Watson test indicated that there was no significant serial autocorrelation in the residuals (*p*-value = 0.1838). A comparison between the predicted and observed values revealed an average deviation of 5.76%, in a range of 0.00%–16.78%. Further statistical insights, including the sum of squares, F-values, and *p*-values for the variables, are reported in Table 6.

**Figure 6:**
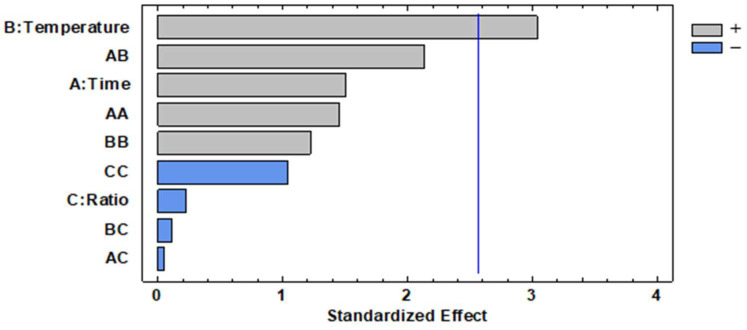
Pareto chart for the synthesis of the different HCDACs using BBD-RSM. The vertical line represents 95% confidence level.

The temperature was found to be the only significant variable influencing yield, with a *p*-value of 0.0288. The results are graphically represented in a Pareto chart (Figure 6), demonstrating a positive effect of temperature on yield. This implies that higher values within the evaluated range correspond to higher yield, as already discussed. Additionally, time exhibited a positive effect, indicating that a greater DS_hydrophobic_ occurs when both time and temperature are at the upper limits of the studied range.

The contributions of each parameter to the yield of the HCDACs preparation are given in Equation (8).

AE = 1.504 − 0.010·Time − 0.003·Temperature − 0.282·Ratio + 5.89 × 10^−4^·Time^2^ + 3.57 × 10^−5^·Time·Temperature − 0.002·Time·Ratio − 1.8 × 10^−4^·Temperature^2^ + 0.015·Temperature·Ratio − 0.217·Ratio^2^ (8)

and the integration was given by Equation (9)

Integration= 14.908 − 0.971·Time − 0.465·Temperature + 4.665·Ratio + 0.013·Time^2^ + 0.010·Time·Temperature − 0.005·Time·Ratio + 0.003·Temperature^2^ − 0.005·Temperature·Ratio − 1.087·Ratio^2^ (9)

**Figure 7:**
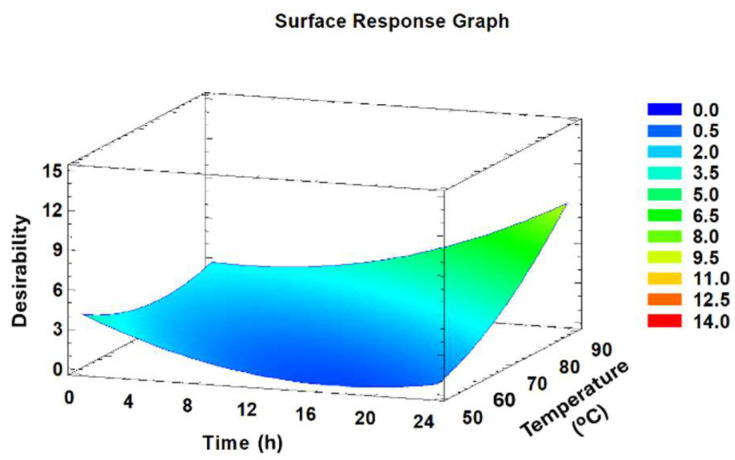
Estimated response surface graph for HCDAC synthesis. Blue colour represents the low desirability, whereas the red colour represents the highest desirability.

The optimisation procedure applied a multi-response analysis to harmonise all reaction conditions towards maximising the DS_hydrophobic_. The outcomes are depicted in a response surface graph (Figure 7), showing that the highest desirability was attained at the upper boundary of the investigated range. Consequently, the optimal conditions were identified as temperature of 90 °C coupled with a reaction time of 24 h.

**Figure 8:**
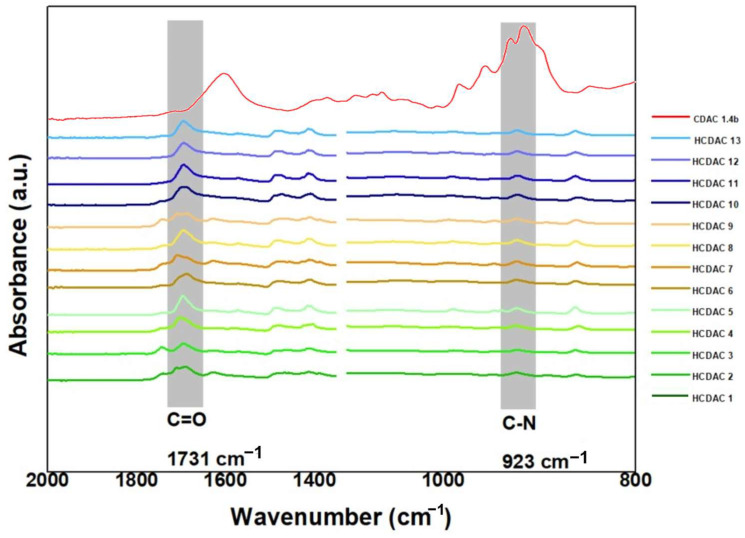
FTIR spectra of HCDACs synthesised at different reaction times (i.e., 3, 13.5, and 24 h) and temperatures (i.e., 50, 70, and 90 °C). For the detailed information on each reaction conditions and composition, see Table 5.

After the optimisation of the synthesis conditions, the chemical structures of the novel HCDACs were characterised using FTIR (Figure 8).

From the esterification of CDAC, a new absorbance peak emerged at 1731 cm^−1^, indicating the stretching vibration mode of the carbonyl group (C=O) from the ester bonds formed between the fatty acid and the OH group linked to the C6 carbon of the CDAC backbone. This confirms the successful esterification of CDAC, and data further suggest a linear increase in carbonyl peak intensity with the increase in the reaction temperature. The absence of the absorption band around 1687 cm^−1^, typically attributed to absorbed water in most HCDACs, further verifies the effectiveness of the reaction conducted in methanol and subsequent washing with hexane [37]. The peak at 925 cm^−1^ can be attributed to the asymmetric stretching of the C–N bond of quaternary ammonium, linked to the cationic group.

**Figure 9:**
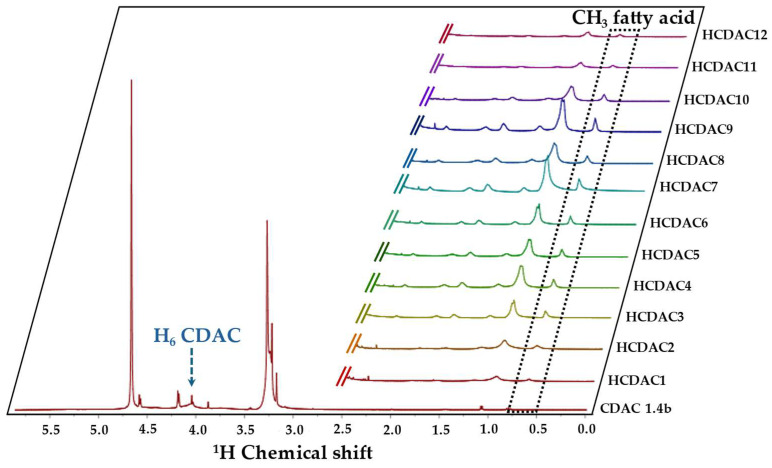
^1^H-RMN spectra of synthesised HCDAC. For the detailed information on each reaction conditions and composition, see Table 5.

The novel HCDAC derivatives were further characterised by ^1^H-NMR (Figure 9). Post-functionalisation, the modified cellulose was insoluble in organic solvents but well-dispersed in deuterated water.

The ^1^H NMR spectra revealed pronounced signals observed in the 3.23–3.29 ppm range, which are attributed to the methyl protons (H_3_C) present in alkylamine fractions, as well as protons associated with the carbon linkage between the alkylamine group and the C=O(NH) amide group, and protons linked to carbon in the HC=N-imine bond. Additionally, a distinctive signal at 4.07 ppm was identified, likely arising from protons connected to the C6 carbon. The relation between the integration of the characteristic peak at 0.82 ppm (attributed to the terminal CH_3_ in fatty acids (see Figure 4)), and the peak at 4.07 ppm (protons in carbon C6 of cellulose), provides a way of estimating the substitution degree of the fatty acids into the CDAC structure.

The combination of NMR and elemental analysis allows for the assessment of the degree of substitution for both the cationic and fatty acid groups, as presented in Table 7.

The calculated DS_hydrophobic_ ranged from 0.09 to 0.66, indicating that reactions occurred under all tested conditions. Notably, at 90 °C, the high DS_hydrophobic_ suggests a superior fatty acid esterification, though such high temperature may also lead to partial degradation of the CDAC chain. Balancing the two DS values (hydrophobic and cationic) is crucial. As the DS_cationic_ decreases, the polymer’s solubility diminishes. From our assays, to achieve a highly water-soluble polymer, the DS_cationic_ should be close to 1, and the DS_hydrophobic_ should be minimised, resulting in a soluble and polyvalent polyelectrolyte, potentially capable of establishing both electrostatic and hydrophobic interactions.

As observed for the cationization step, data also indicate that increasing the reaction temperature improves the DS_hydrophobic_ of the resulting HCDACs. However, the nitrogen content in the structure decreases with further higher temperatures (data included in Supplementary Materials), likely due to the competition between the esterification reaction and the partial hydrolysis of the cationic groups, as water, a byproduct of the reaction, can trigger hydrolysis. Moisture in the reaction medium is thus detrimental to the process. For samples HCDAC 7 and HCDAC 8, the DS_hydrophobic_ values were 0.660 and 0.317, respectively, yet no significant changes in DS_cationic_ were observed. Thus, 90 °C was considered the higher temperature limit wherein the esterification process can still occur effectively without compromising the cationic modification.

**Figure 10:**
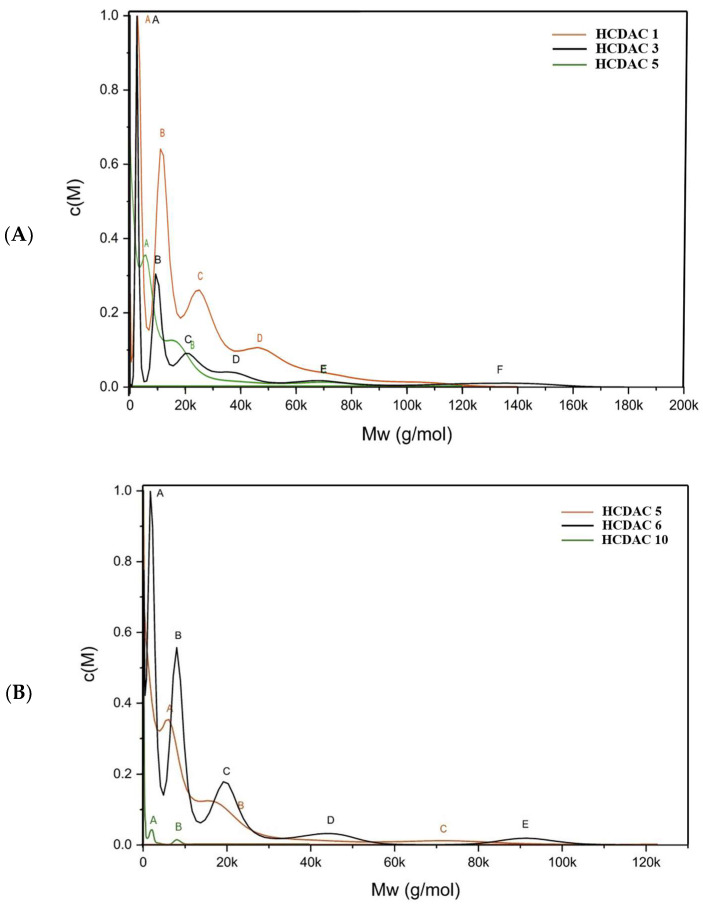
Molecular mass distribution for selected HCDAC derivatives: (**A**) with different fatty acid/CDAC ratios and (**B**) with different reaction temperatures. For detailed information on each reaction conditions and composition, see Table 5. The letters A–F refer to peaks identified on each sample (please see Supplementary Figure S2, for more details)

After the structural characterisation provided by FTIR and ^1^H NMR, the molecular weight of the novel HCDAC derivatives was assessed by analytical ultracentrifugation. In Figure 10, HCDACs were prepared with different fatty acid/CDAC ratios and temperatures. The molecular mass distributions for all HCDAC prepared are shown in the Supplementary Materials.

The increase in the DS_hydrophobic_ (Figure 10A) reveals a progressive decrease in the molecular weight distributions width. From HCDAC1 to HCDAC5, there is a consistent decrease in the width of the distribution. However, the HCDAC3 presents a wider molecular weight distribution, which could be related to the lower DS_cationic_ observed and consequently to a possible higher aggregation of the polymer chains resulting in a wider distribution. Conversely, as temperature increases (Figure 10B), there is an increase in the width of the distribution of molecular weights. Again, this could be attributed to the higher DS_hydrophobic_ and lower cationicity of the cellulose derivatives, which may trigger the formation of aggregates, rendering the molecular weight distribution wider. Overall, data suggest that elevated temperatures and higher levels of DS_hydrophobic_ may be prone to the observable increase in the distribution width.

The HCDACs were further characterised regarding their zeta potential and effect on the water surface tension (Table 8).

The introduction of hydrophobic modifications considerably enhances the surface activity of the polymers, as evidenced by the notable decrease in surface tension with increasing DS_hydrophobic_. These novel HCDACs exhibit a pronounced tendency to migrate towards interfaces, demonstrating efficacy even at low concentrations. Conversely, the zeta-potential increases with hydrophobic substitution. This can be attributed to the higher exposure of the cationic moieties to the aqueous medium upon chain molecular rearrangement to minimise the energetically unfavourable exposure of the hydrophobic groups to the aqueous medium [38]. It is important to note that zeta-potential serves as an indicator of charged functional groups on the particle surface, the value of which can depend on various factors, such as pH, ionic strength of the liquid phase, porosity and roughness of the particle, conductivity of the medium, and aggregation state. By comparing the DS_hydrophobic_ of the different synthesised polyelectrolytes with their estimated hydrodynamic radius, we observed, tendentially, a slight increase in the hydrodynamic radius as the DS_hydrophobic_ increases. This is because the fatty acid chains promote polymer aggregation in aqueous medium, thereby increasing the hydrodynamic radius. This observation aligns with findings in the literature, which indicate that hydrophobic interactions among fatty acid chains enhance the overall size and stability of the polymer aggregates [39].

## 4. Conclusions

This work elucidates the rather sustainable procedures to craft an extensive library of novel cellulose-based derivatives with tunned hydrophobic and cationic moieties. Periodate oxidation was selected for its efficiency and adaptability across diverse modifications to render the cellulose chain more reactive to the cationization step. A series of DACs with varying aldehyde group contents were synthesised, and it was found that a higher sodium periodate concentration in the reaction resulted in a superior aldehyde content.

Following the establishment of the periodate oxidation protocol, a sequence of cationization experiments was conducted for each DAC produced utilising GT. Adjusting the GT/aldehyde group ratio yielded varied charge densities, with reactions performed at 70 °C for 1 h. The results reveal a direct correlation between the amount of GT reagent used and the resultant charge density. Elemental analysis, specifically nitrogen determination, was used to obtain the DS_cationic_. A direct correlation between zeta potential and DS_cationic_ was not observed, which can be due to an increased intensity of the interaction of the charged cationic groups with the counterions in the polymers with higher DS_cationic_.

Finally, HCDACs were successfully synthesised by modifying CDAC with fatty acids extracted from vegetable oil with a lipase-assisted method. The extracted fatty acids were fully characterised, and the temperature, CDAC-to-fatty acid ratio, and reaction time were found to affect the HCDAC formation. In particular, the DS_hydrophobic_ was particularly susceptible to increase at higher temperatures (i.e., 90 °C). Further characterisation employing FTIR and ^1^H NMR confirmed the successful hydrophobic modification of CDAC by esterification of OH in the C6 position with the extracted fatty acids. Regarding the final yield, the optimal reaction conditions were identified as 24 h reaction time at 90 °C, with a ratio between fatty acid and CDAC 1.4b of 1:2.

The synthesised HCDACs were found to be generally soluble in water at room temperature. This comprehensive study proves that highly charged, cellulose-based cationic and hydrophobic polyelectrolytes can be tailored through an adequate set of modifications: periodate oxidation, cationization, and hydrophobisation. As a result, it was possible to produce a diverse library of HCDACs with distinct charge densities, rendering them suitable for use in different applications, including as effective flocculation agents in wastewater treatment. Ongoing work is evaluating how the different HCDACs here developed can selectively flocculate pollutants of different features. By considering the use of Acasia wood residues, this work also contributes to valorising forestry residues from an invasive species in Portugal with the development of multi-functional cellulose derivatives with added value.

## Figures and Tables

**Table 1:** Composition of native Acacia wood and extracted cellulose.

Composition (%*w*/*w*)			
	Lignin	Cellulose	Hemicellulose
Acacia wood	22.20 (±0.30)	47.41 (±0.90)	18.79 (±0.25)
Cellulose-rich fraction	1.48 (±0.16)	94.80 (±3.13)	2.84 (±0.63)

**Table 2:** Reaction conditions for extraction fatty acids from vegetable oil.

	0.25% (*w*/*v*) Lipase			0.50% (*w*/*v*) Lipase		
Time (h)	1	2	3	1	2	3
Vegetable oil (g)	50	50	50	50	50	50
Water (mL)	50	50	50	50	50	50
Fatty acid yield (%)	84	79	87	63	51	44

**Table 3:** Reaction conditions for the synthesis of DAC from cellulose extracted from Acacia wood.

Sample	Suspension (% *w*/*w*)	NaIO_4_ (mol·mol^−1^)	Aldehyde. Content (mmol·g^−1^)	t (h)	T (°C)	DS_DAC_
DAC 0.3b	4	0.5	1.50	3	70	0.30
DAC 1b	4	1.5	8.25	3	70	1.51
DAC 1.2b	4	2.0	9.60	3	70	1.80
DAC 1.4b	4	2.5	10.35	3	70	1.96
DAC 1.8b	4	3.0	12.40	3	70	2.44

**Table 4:** Reaction conditions for the synthesis of CDAC from DAC.

Sample	Ald. Content (mmol·g^−1^)	DS_DAC_	GT/Ald (mol/mol)	t (min)	T (°C)	DS_CDAC_	ζ-Potential (mV)
CDAC 0.3b	1.50	0.30	0.5	90	70	0.3	+36.23 (±4.98)
CDAC 1b	8.25	1.51	1.0	90	70	1	+33.88 (±2.59)
CDAC 1.2b	9.60	1.80	1.5	90	70	1.2	+33.20 (±1.10)
CDAC 1.4b	10.35	1.96	1.5	90	70	1.4	+30.73 (±0.38)
CDAC 1.8b	12.40	2.44	2.0	90	70	1.8	+24.28 (±4.23)

**Table 5:** Reaction conditions for synthesising hydrophobised cationic cellulose from Acacia wood according to BBD-RSM.

Experiment	K_2_CO_3_ (g)	MeOH (mL)	Ratio CDAC/Fatty Acid (mol·mol^−1^)	Time (h)	Temp. (°C)	HCDAC Yield (%)
HCDAC 1	0.01	10	1:1	3	70	32.9
HCDAC 2	0.01	10	1:1	24	70	35.8
HCDAC 3	0.01	10	1:3	24	70	37.5
HCDAC 4	0.01	10	1:3	3	70	32.0
HCDAC 5	0.01	10	1:2	13.5	70	33.4
HCDAC 6	0.01	10	1:2	3	90	47.2
HCDAC 7	0.01	10	1:2	24	90	47.2
HCDAC 8	0.01	10	1:3	13.5	90	45.3
HCDAC 9	0.01	10	1:1	13.5	90	42.1
HCDAC 10	0.01	10	1:2	3	50	27.5
HCDAC 11	0.01	10	1:2	24	50	22.6
HCDAC 12	0.01	10	1:1	13.5	50	22.5
HCDAC 13	0.01	10	1:3	13.5	50	24.0

**Table 6:** Results from the BBD–RSM applied to the synthesis of the HCDACs.

Variable	Sum of Squares	F-Value	*p*-Value
Time (A)	9.12	2.28	0.1917
Temperature (B)	36.98	9.24	0.0288
Ratio CDAC/fatty acid (C)	0.21	0.05	0.8274
AA	8.51	2.12	0.2047
AB	18.19	4.54	0.0863
AC	0.01	0.00	0.9602
BB	6.03	1.51	0.2744
BC	0.06	0.01	0.9111
CC	4.36	1.09	0.3444
Total error	20.02		

**Table 7:** DS of HCDACs at different reaction conditions.

Polymers	Time (h)	Temperature (°C)	Mass Ratio (Fatty Acid/CDAC)	DS_hydrophobic_	DS_cationic_
Cationic 1.4b	-	-	-	-	1.4
HCDAC 1	3.0	70	1:1	0.090	1.09
HCDAC 2	24.0		1:1	0.103	0.97
HCDAC 3	24.0		1:3	0.130	0.96
HCDAC 4	3.0		1:3	0.117	0.87
HCDAC 5	13.5		1:2	0.193	1.04
HCDAC 6	3.0	90	1:2	0.240	0.94
HCDAC 7	24.0		1:2	0.660	0.99
HCDAC 8	13.5		1:3	0.317	0.96
HCDAC 9	13.5		1:1	0.263	0.92
HCDAC 10	3.0	50	1:2	0.197	1.11
HCDAC 11	24.0		1:2	0.153	1.13
HCDAC 12	13.5		1:1	0.227	1.16

**Table 8:** Surface tension, ζ-potential, and average diameter of the HCDACs in solution.

Cellulose Derivative	Surface Tension (mN·m^−1^)	ζ-Potential (mV)	Z-Av. Diameter (nm)
CDAC	66.5 (± 0.7)	30.7 (± 0.4)	18.7
HCDAC 1	27.2 (± 1.2)	52.2 (± 0.7)	110.8
HCDAC 2	24.7 (± 0.9)	59.8 (± 2.0)	99.7
HCDAC 3	24.5 (± 0.2)	60.3 (± 3.8)	92.6
HCDAC 4	26.5 (±0.3)	59.6 (± 1.5)	99.0
HCDAC 5	21.1 (±0.2)	57.2 (± 1.0)	89.2
HCDAC 6	22.5 (±0.3)	64.7 (± 1.4)	70.7
HCDAC 7	21.2 (±0.7)	67.3 (± 2.1)	94.7
HCDAC 8	21.3 (±0.4)	64.2 (± 1.7)	90.7
HCDAC 9	24.7 (±0.6)	68.9 (± 1.4)	90.7
HCDAC 10	42.0 (±0.3)	44.7 (± 2.7)	70.1
HCDAC 11	46.0 (±0.1)	49.3 (± 0.9)	79.2
HCDAC 12	30.2 (±0.4)	45.3 (± 3.0)	81.9

## Data Availability

Data are contained within the article or Supplementary Material.

## Supplementary Materials

The following supporting information can be downloaded at: https://www.mdpi.com/article/10.3390/polym16223105/s1, Figure S1: (1): (ρ-ρ0)–c plot to determine the density of different hydrophobically modified cationic cellulose (HCDAC) solute, which is derived from the measurements with a Kratky density (see Equation (7)) in water at 20 °C. (A) for the determined density increment ∂ρ⁄∂c of 0.241716, the density of HCDAC1 is 1.316 g/mL. (B) for the determined density increment ∂ρ⁄∂c of 0.178003, the density of HCDAC2 is 1.214 g/mL. (2) (continued from previous page): (C) for the determined density increment ∂ρ⁄∂c of 0.248054, the density of HCDAC3 is 1.328 g/mL. (D) for the determined density increment ∂ρ⁄∂c of 0.211251, the density of HCDAC4 is 1.266 g/mL. (3) (continued from previous page): (E) for the determined density increment ∂ρ⁄∂c of 0.25339, the density of HCDAC5 is 1.337 g/mL. (F) for the determined density increment ∂ρ⁄∂c of 0.237371, the density of HCDAC6 is 1.309 g/mL. (4) (continued from previous page): (G) for the determined density increment ∂ρ⁄∂c of 0.24907, the density of HCDAC7 is 1.329 g/mL. (H) for the determined density increment ∂ρ⁄∂c of 0.253973, the density of HCDAC8 is 1.338 g/mL. (5) (continued from previous page): (I) for the determined density increment ∂ρ⁄∂c of 0.244034, the density of HCDAC11 is 1.320 g/mL. (J) for the determined density increment ∂ρ⁄∂c of 0.25268, the density of HCDAC12 is 1.336 g/mL.; Figure S2: (1): Normalized calculated molar mass distributions c(M) for hydrophobically modified cationic cellulose polymers. The table in the plot shows the “PEAK INFO” of the different peaks in c(M) distribution using a high-resolution sedimentation coefficient distribution c(s) in SEDFIT program. (A) for HCDAC1 with the determined density of 1.316 g/mL and a best-fit averaged friction ration of 1.116. (B) for HCDAC2 with the determined density of 1.214 g/mL and a best-fit averaged friction ration of 1.182. (2): (continued from previous page): (C) for HCDAC3 with the determined density of 1.328 g/mL and a best-fit averaged friction ration of 1.183. (D) for HCDAC4 with the determined density of 1.266 g/mL and a best-fit averaged friction ration of 1.153. (3): (continued from previous page): (E) for HCDAC5 with the determined density of 1.337 g/mL and a best-fit averaged friction ration of 1.150. (F) for HCDAC6 with the determined density of 1.309 g/mL and a best-fit averaged friction ration of 1.139. (4): (continued from previous page): (G) for HCDAC7 with the determined density of 1.329 g/mL and a best-fit averaged friction ration of 1.157. (H) for HCDAC8 with the determined density of 1.338 g/mL and a best-fit averaged friction ration of 1.186. (5): (continued from previous page): (I) for HCDAC11 with the determined density of 1.320 g/mL and a best-fit averaged friction ration of 1.187. (J) for HCDAC12 with the determined density of 1.336 g/mL and a best-fit averaged friction ration of 1.187.
