# Supplementary material for: Customising Sustainable Bio-Based Polyelectrolytes: Introduction of Charged and Hydrophobic Groups in Cellulose
Source: Polymers (Basel). 2024 Nov 5;16(22):3105. doi: 10.3390/polym16223105 (PMC11597907; doi:10.3390/polym16223105)
Supplement: Supplementary file 1 [file polymers-16-03105-s001.zip › polymers-3263348-supplementary.pdf]

Supplementary Material

Customizing sustainable bioflocculants: introduction of charged and hydrophobic groups in cellulose.

Solange Magalhães, María José Aliaño-González, Pedro Cruz, Rose Rosenberg, Magnus Norgren, Luís Alves, Bruno Medronho and Maria da Graça Rasteiro

Data for $DS_{hydrophobic}$ calculations:

Experiment	Ratio CDAC/fatty acid (mol mol^{-1})	Peak 4.07	Peak 0.82	$DS_{hydrophobic}$
**HCDAC 1**	1:1	1	0.27	0.090
**HCDAC 2**	1:1	1	0.31	0.103
**HCDAC 3**	1:3	1	0.39	0.130
**HCDAC 4**	1:3	1	0.35	0.117
**HCDAC 5**	1:2	1	0.58	0.193
**HCDAC 6**	1:2	1	0.72	0.240
**HCDAC 7**	1:2	1	1.98	0.660
**HCDAC 8**	1:3	1	0.95	0.317
**HCDAC 9**	1:1	1	0.79	0.263
**HCDAC 10**	1:2	1	0.59	0.197
**HCDAC 11**	1:2	1	0.46	0.153
**HCDAC 12**	1:1	1	0.68	0.227

1

(A) HCDAC1

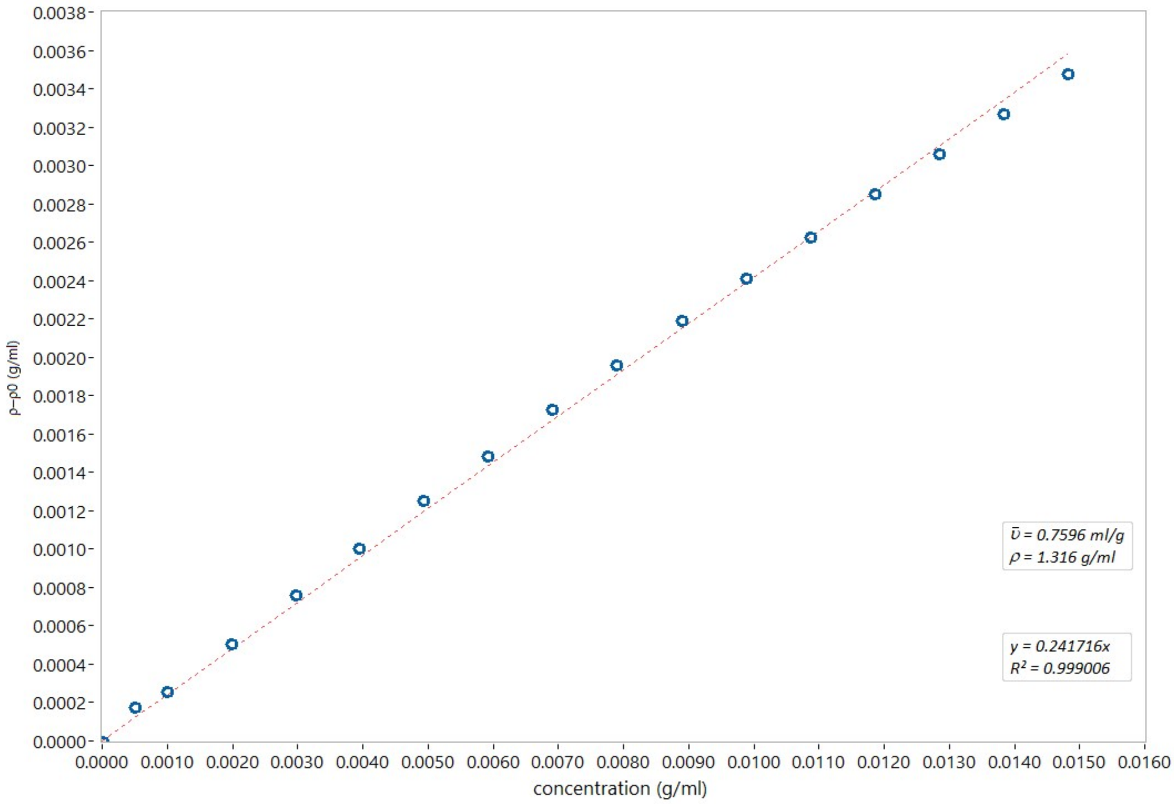

(B) HCDAC2

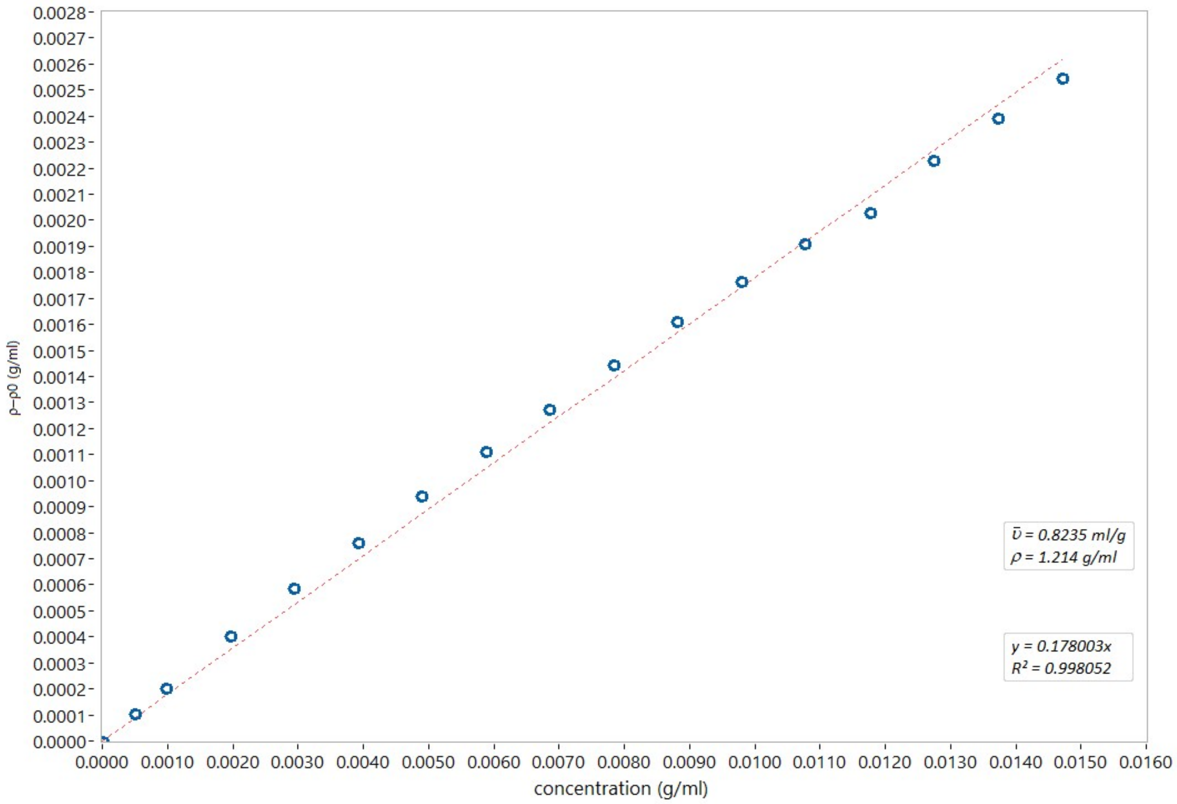

2

(C) HCDAC3

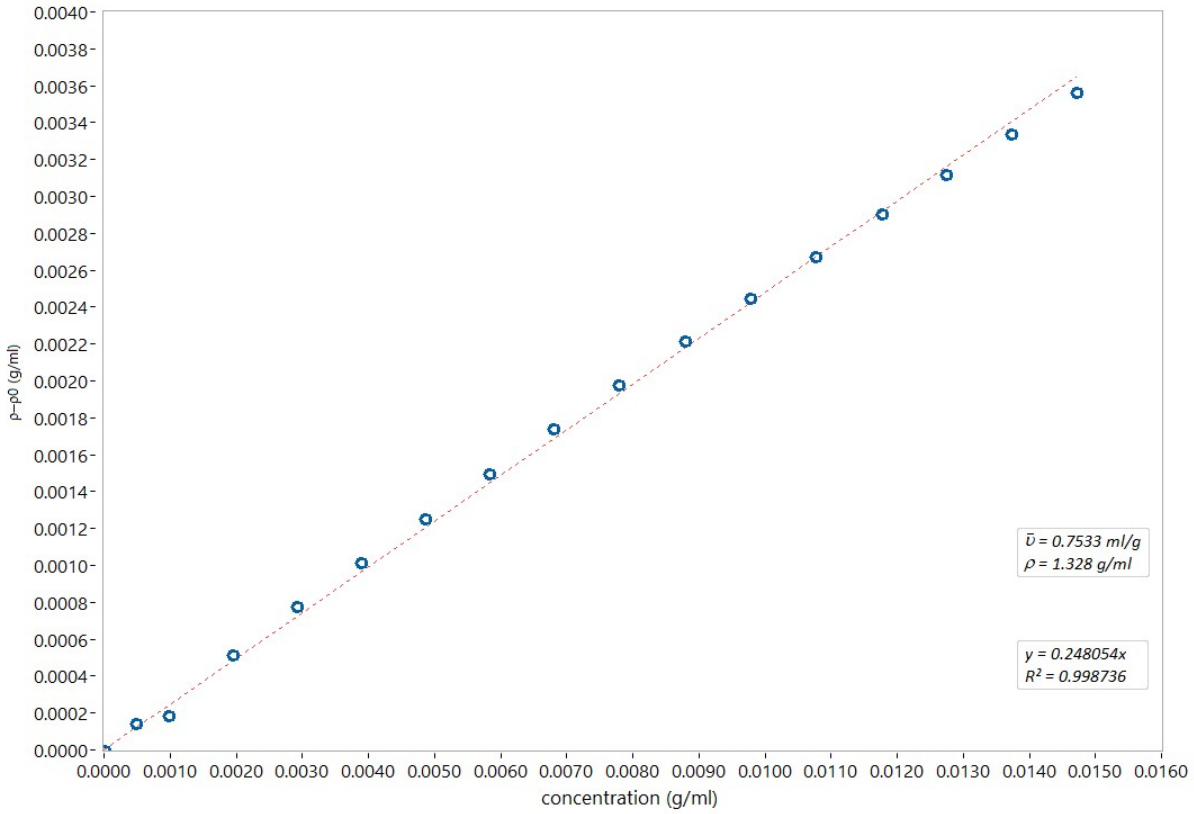

(D) HCDAC4

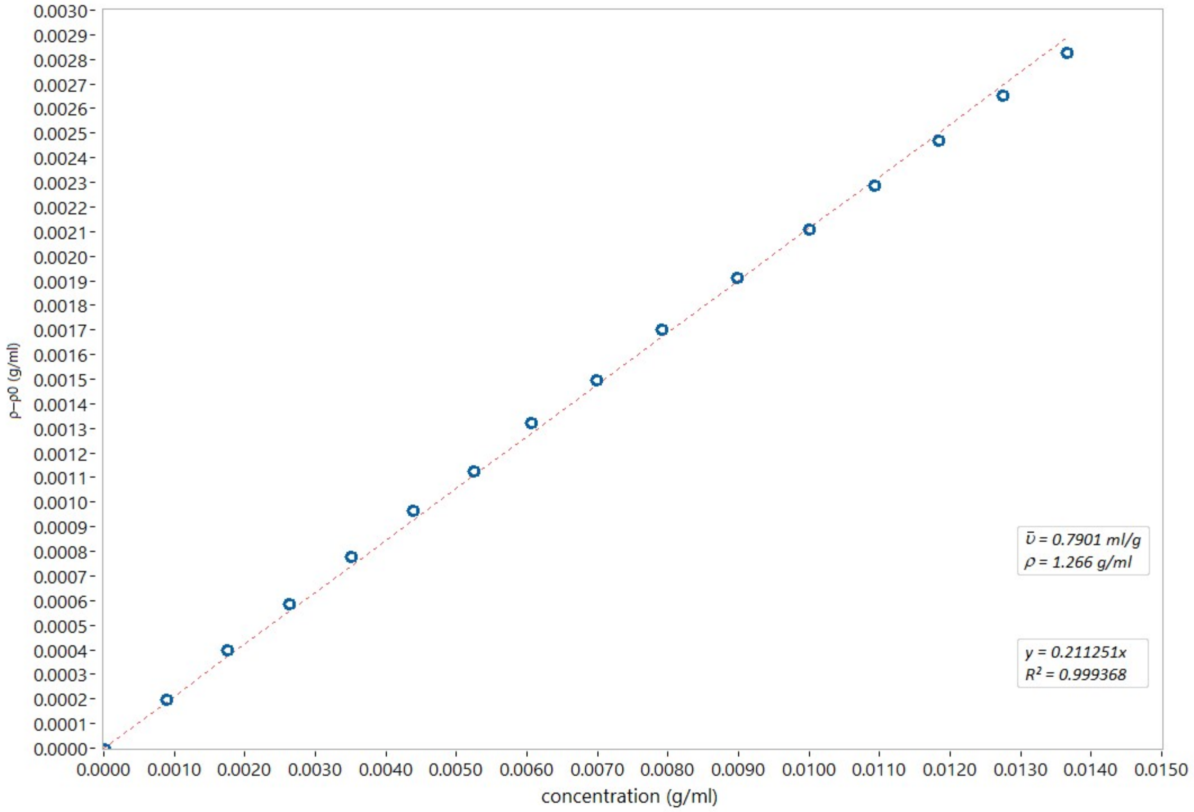

3

(E) HCDAC5

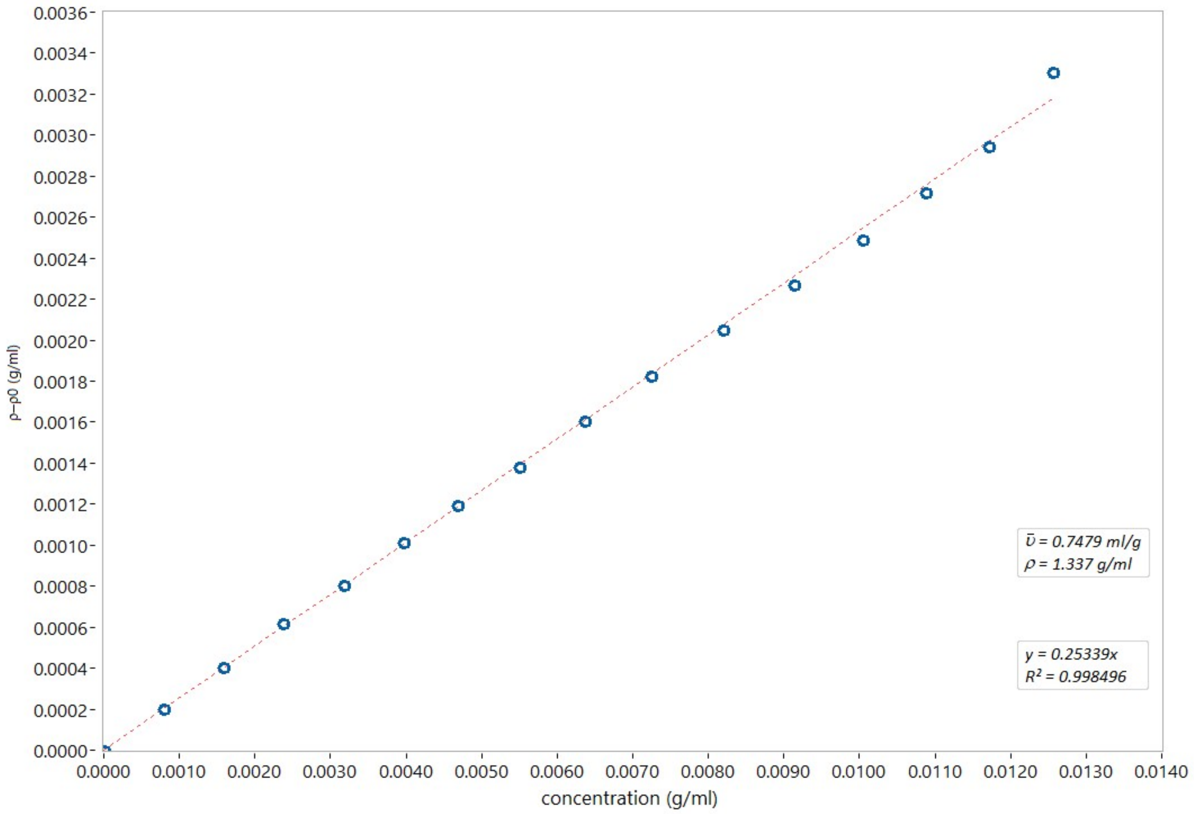

(F) HCDAC6

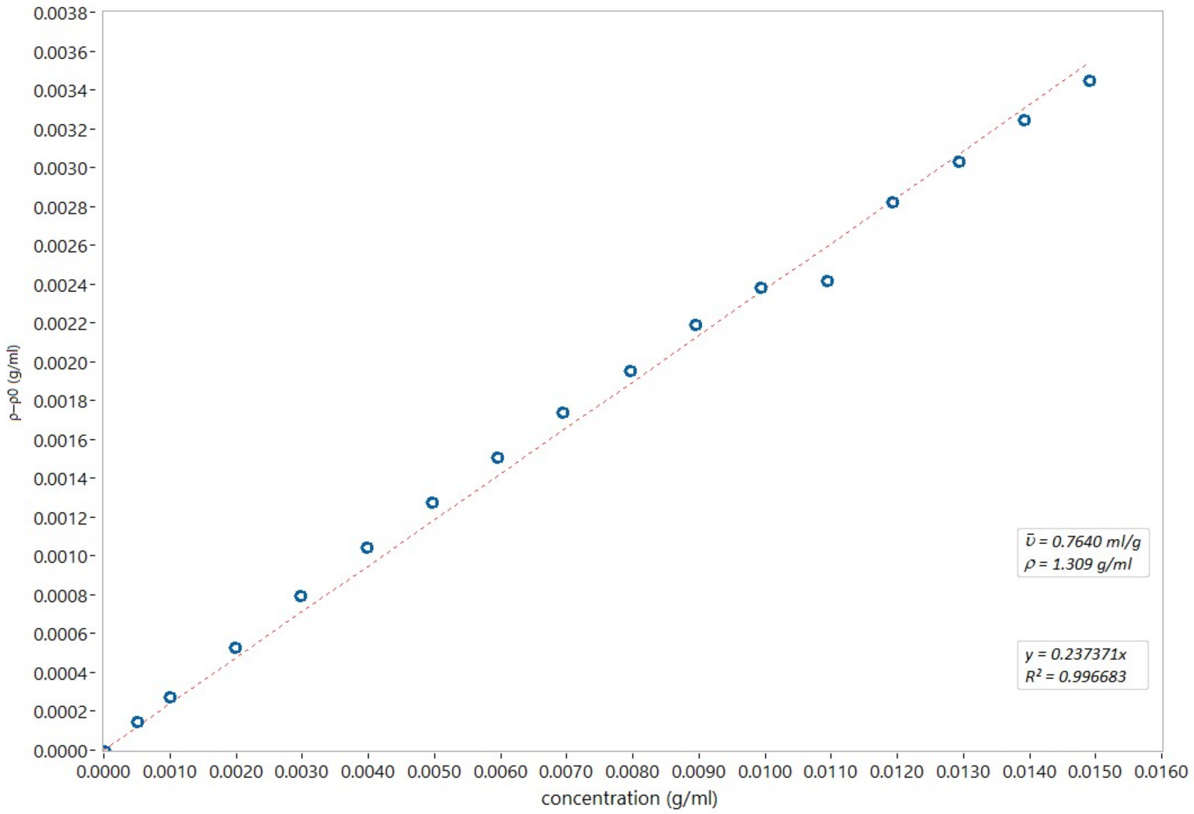

4

(G) HCDAC7

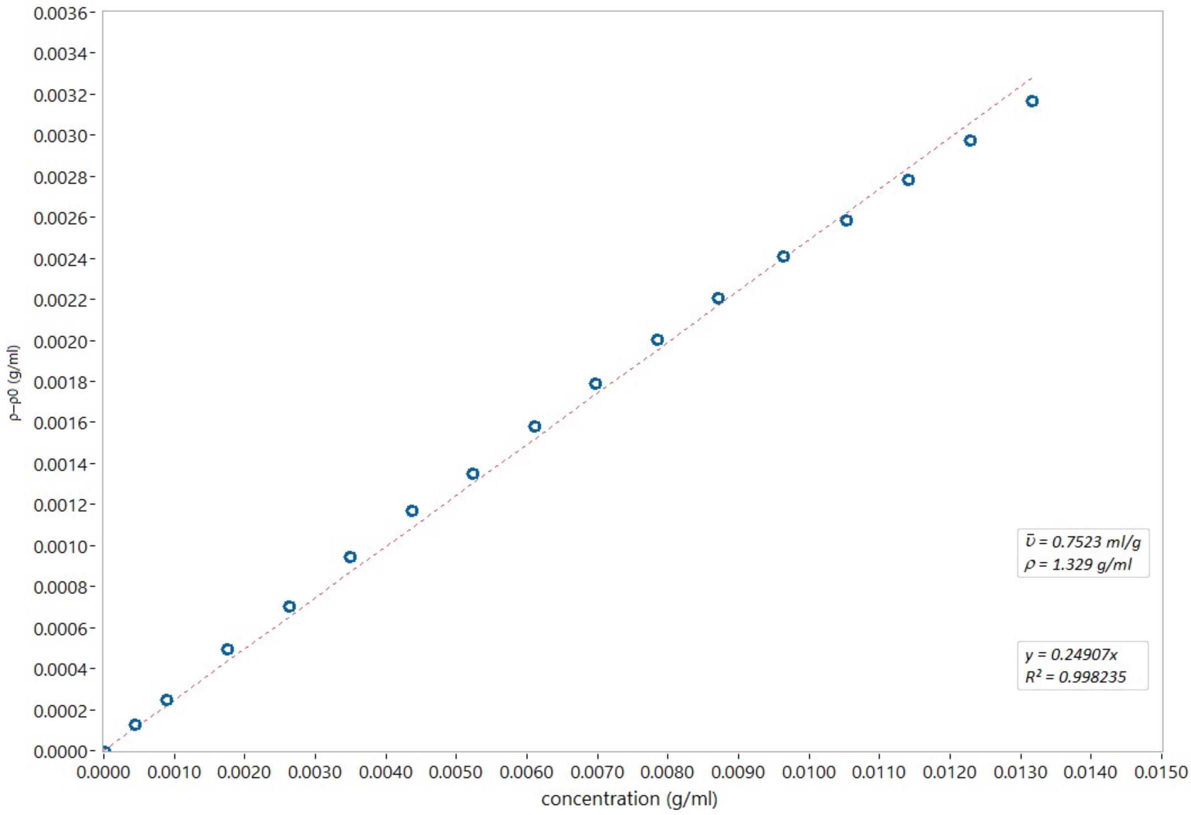

(H) HCDAC8

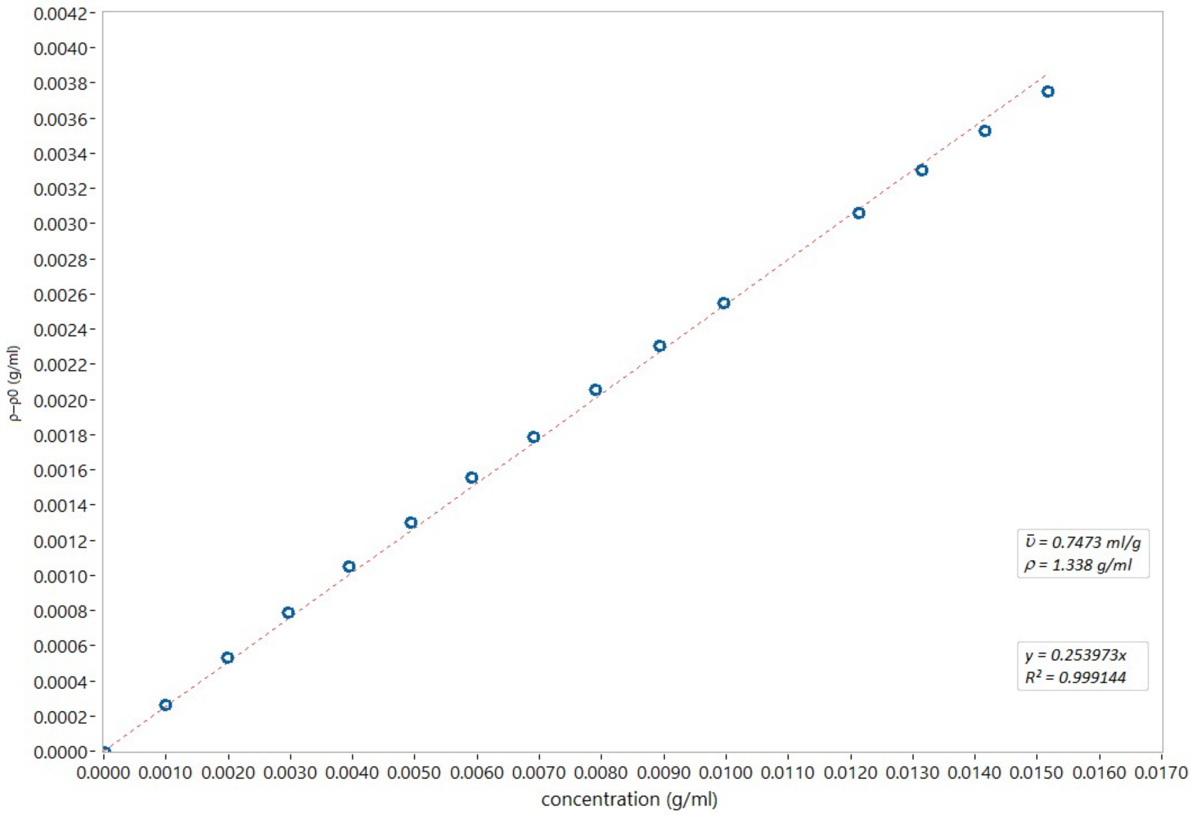

5

(I) HCDAC11

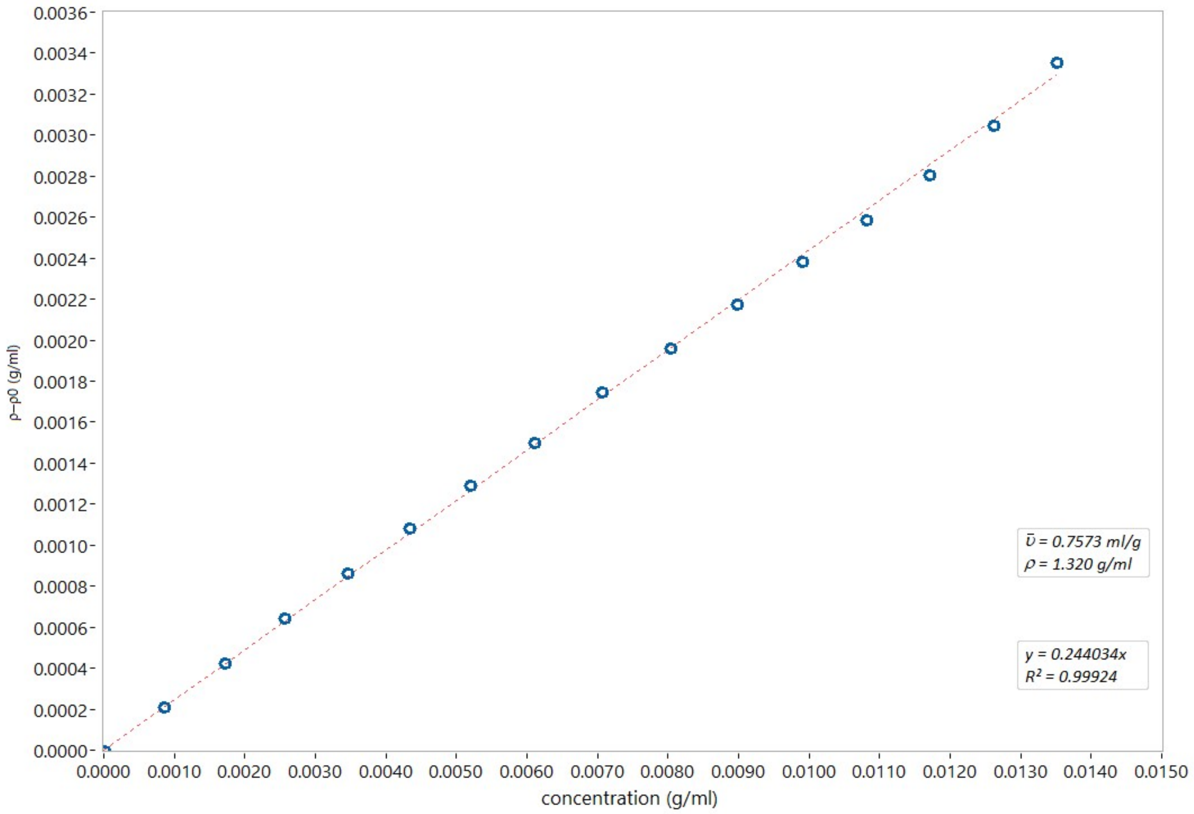

(J) HCDAC12

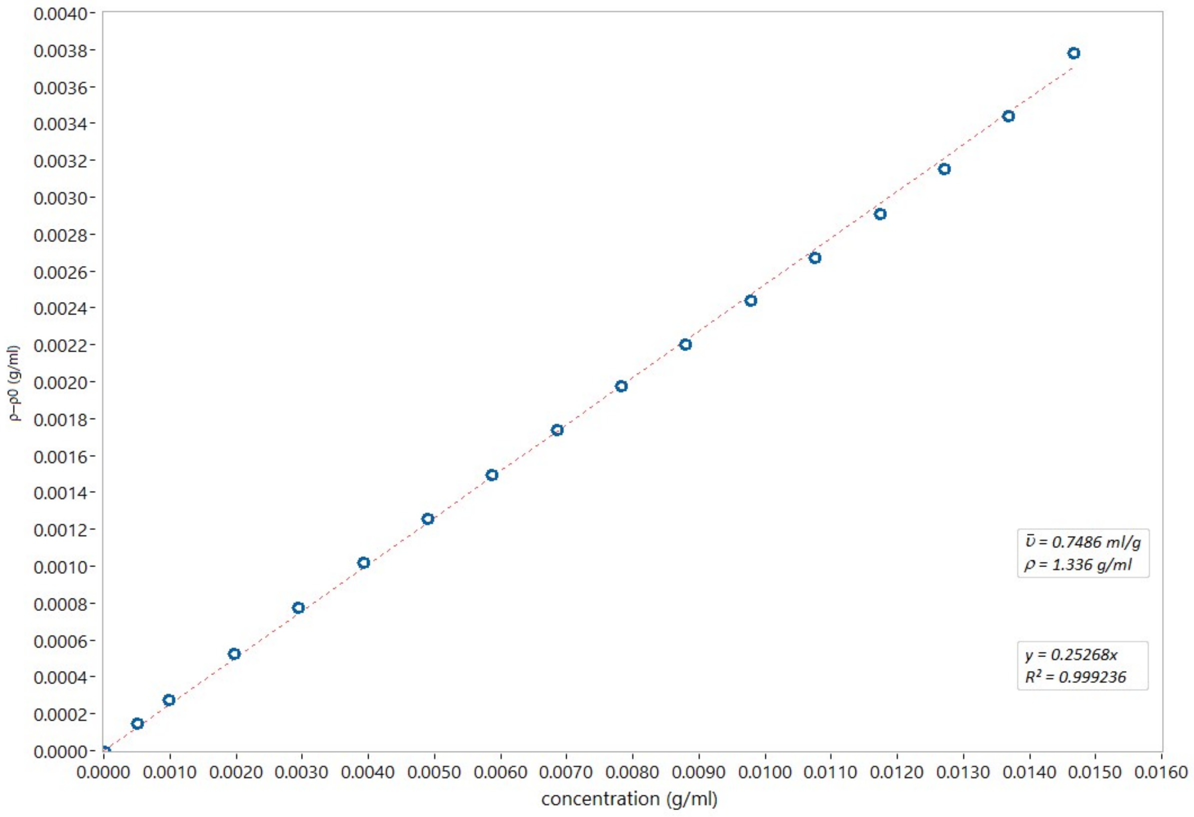

Figure S1. (1): (ϱ-ϱ0) – c plot to determine the density of different hydrophobically modified cationic cellulose (HCDAC) solute, which is derived from the measurements with a Kratky density (see Eq. 7) in water at 20 °C. **(A)** for the determined density increment ∂ϱ/∂c of 0.241716, the density of HCDAC1 is

1.316 g/ml. **(B)** for the determined density increment $\partial\varrho/\partial c$ of 0.178003, the density of HCDAC2 is 1.214 g/ml. (**2) (continued from previous page): (C)** for the determined density increment $\partial\varrho/\partial c$ of 0.248054, the density of HCDAC3 is 1.328 g/ml. **(D)** for the determined density increment $\partial\varrho/\partial c$ of 0.211251, the density of HCDAC4 is 1.266 g/ml. (**3) (continued from previous page): (E)** for the determined density increment $\partial\varrho/\partial c$ of 0.25339, the density of HCDAC5 is 1.337 g/ml. **(F)** for the determined density increment $\partial\varrho/\partial c$ of 0.237371, the density of HCDAC6 is 1.309 g/ml. (**4) (continued from previous page): (G)** for the determined density increment $\partial\varrho/\partial c$ of 0.24907, the density of HCDAC7 is 1.329 g/ml. **(H)** for the determined density increment $\partial\varrho/\partial c$ of 0.253973, the density of HCDAC8 is 1.338 g/ml. (**5) (continued from previous page): (I)** for the determined density increment $\partial\varrho/\partial c$ of 0.244034, the density of HCDAC11 is 1.320 g/ml. **(J)** for the determined density increment $\partial\varrho/\partial c$ of 0.25268, the density of HCDAC12 is 1.336 g/ml.

1

(A)

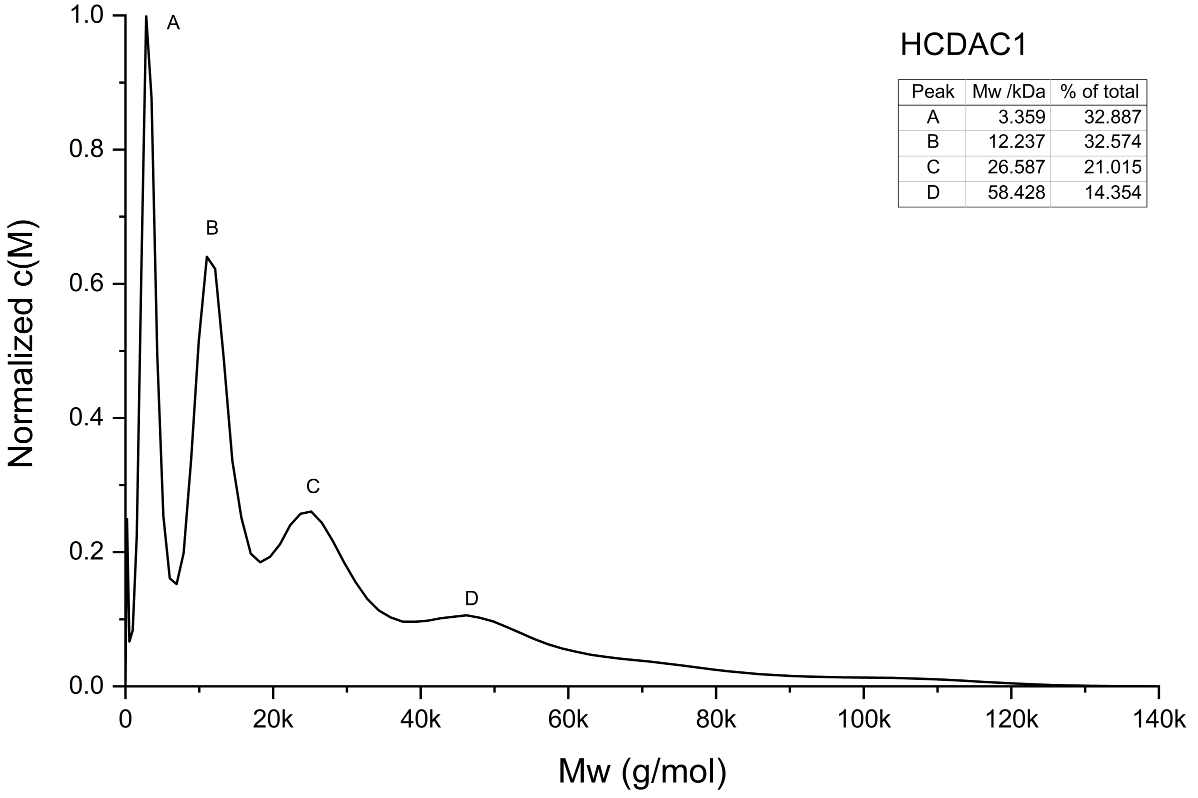
HCDAC1
Peak | Mw /kDa | % of total
A | 3.359 | 32.887
B | 12.237 | 32.574
C | 26.587 | 21.015
D | 58.428 | 14.354
A
B
C
D
Normalized c(M)
Mw (g/mol)
0
20k
40k
60k
80k
100k
120k
140k
0.0
0.2
0.4
0.6
0.8
1.0

(B)

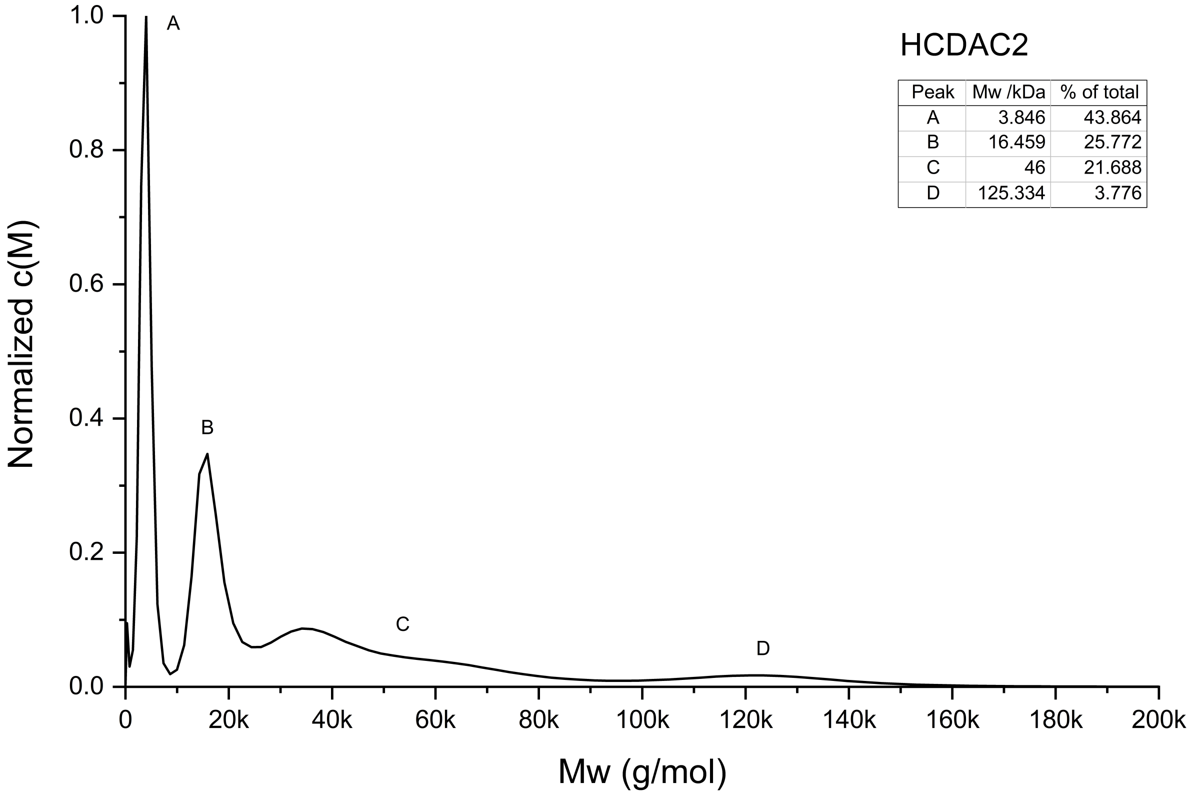
HCDAC2
Peak | Mw /kDa | % of total
A | 3.846 | 43.864
B | 16.459 | 25.772
C | 46 | 21.688
D | 125.334 | 3.776
A
B
C
D
Normalized c(M)
Mw (g/mol)
0
20k
40k
60k
80k
100k
120k
140k
160k
180k
200k
0.0
0.2
0.4
0.6
0.8
1.0

(C)

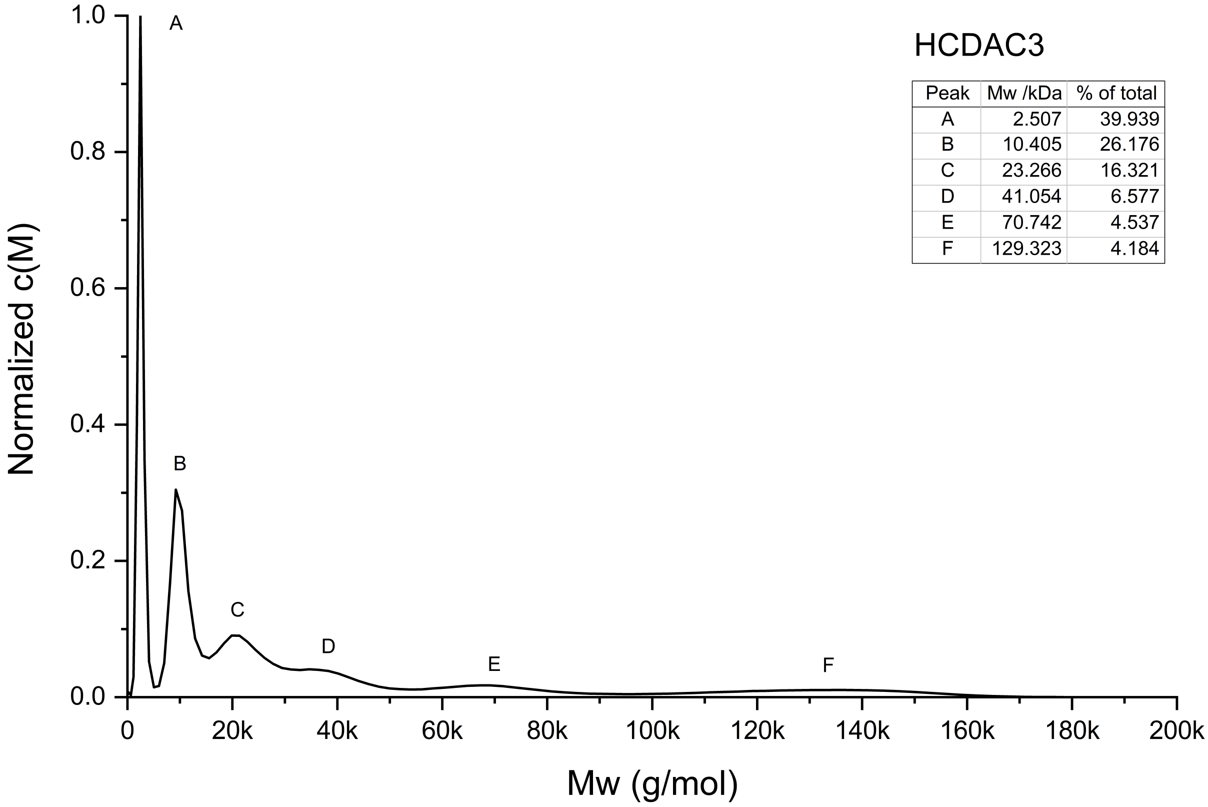

Peak	Mw /kDa	% of total
A	2.507	39.939
B	10.405	26.176
C	23.266	16.321
D	41.054	6.577
E	70.742	4.537
F	129.323	4.184

(D)

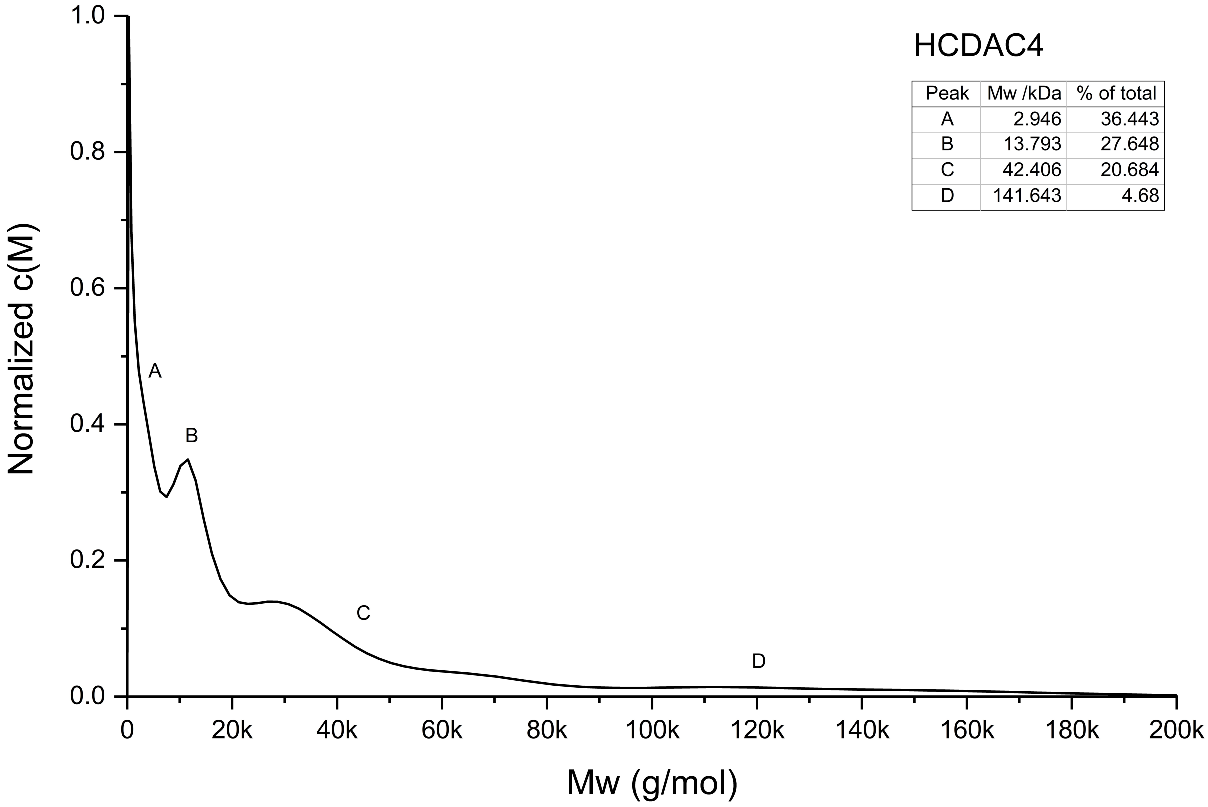

Peak	Mw /kDa	% of total
A	2.946	36.443
B	13.793	27.648
C	42.406	20.684
D	141.643	4.68

3

(E)

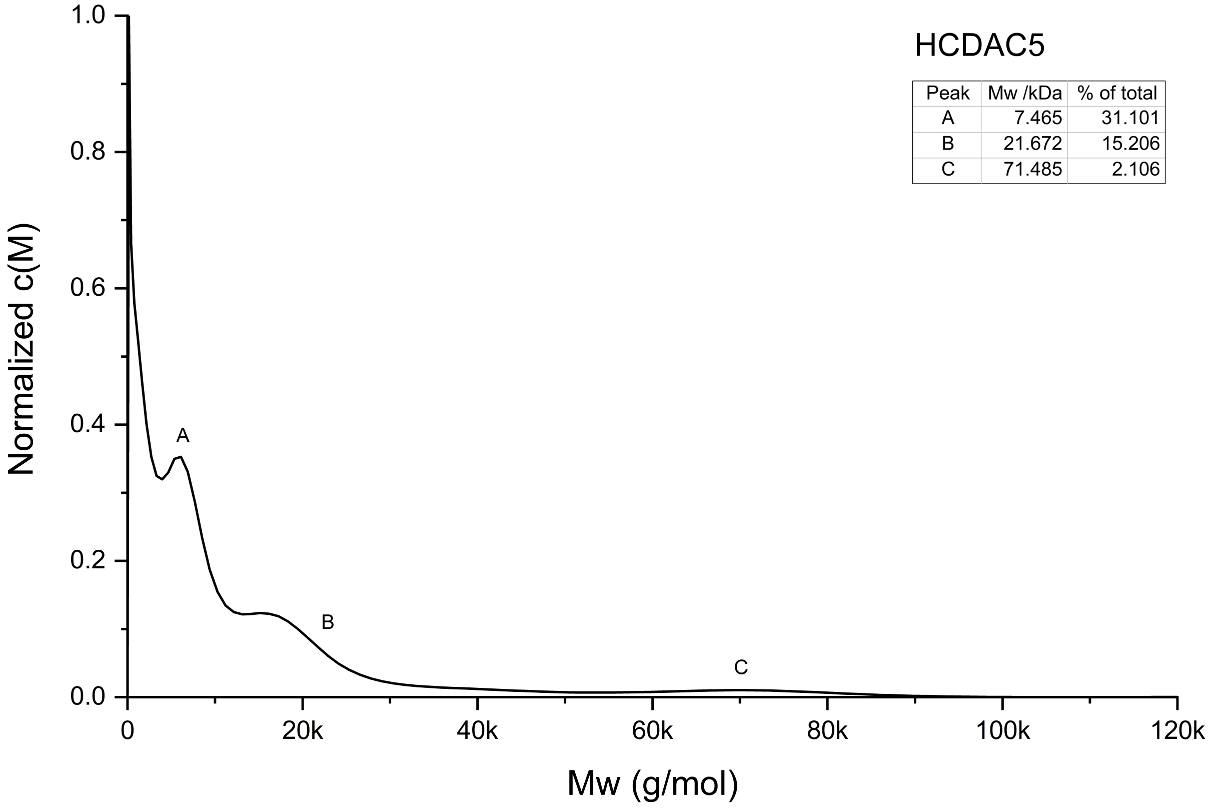
HCDAC5
Peak | Mw /kDa | % of total
A | 7.465 | 31.101
B | 21.672 | 15.206
C | 71.485 | 2.106
A
B
C
Normalized c(M)
Mw (g/mol)

(F)

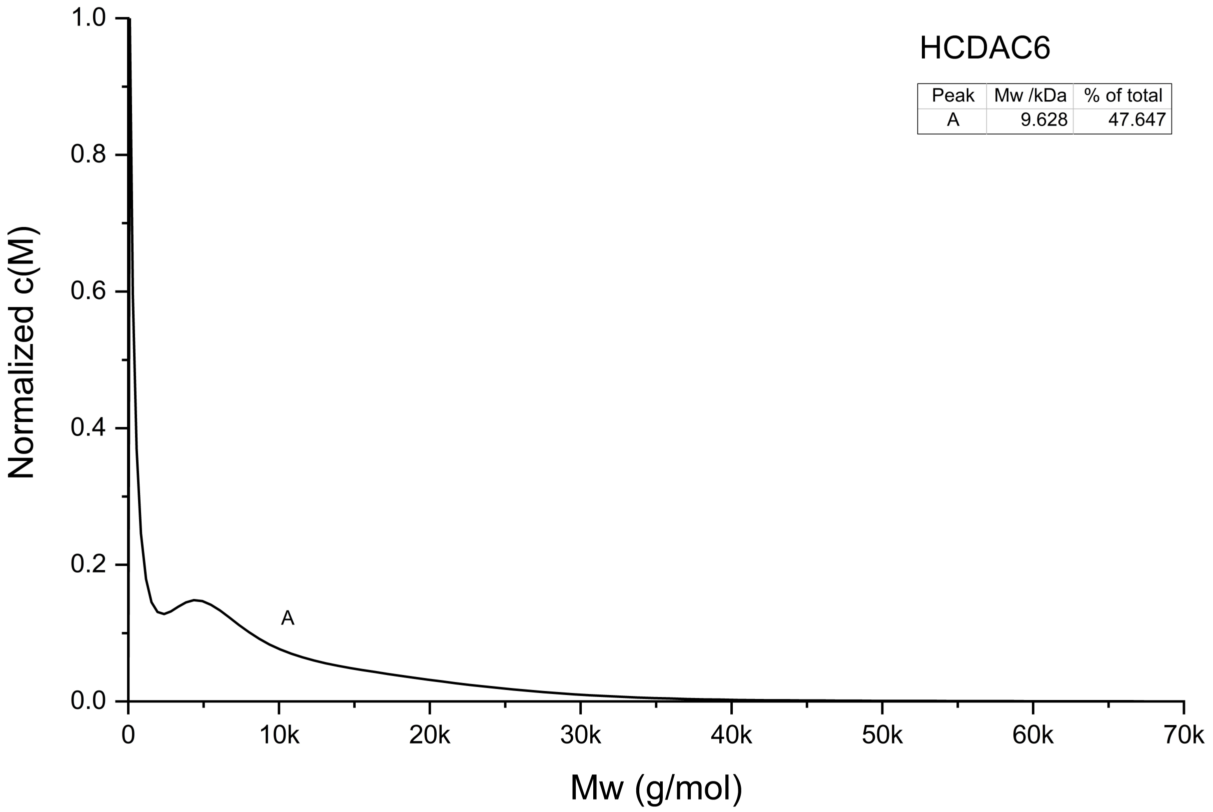
HCDAC6
Peak | Mw /kDa | % of total
A | 9.628 | 47.647
A
Normalized c(M)
Mw (g/mol)

4

(G)

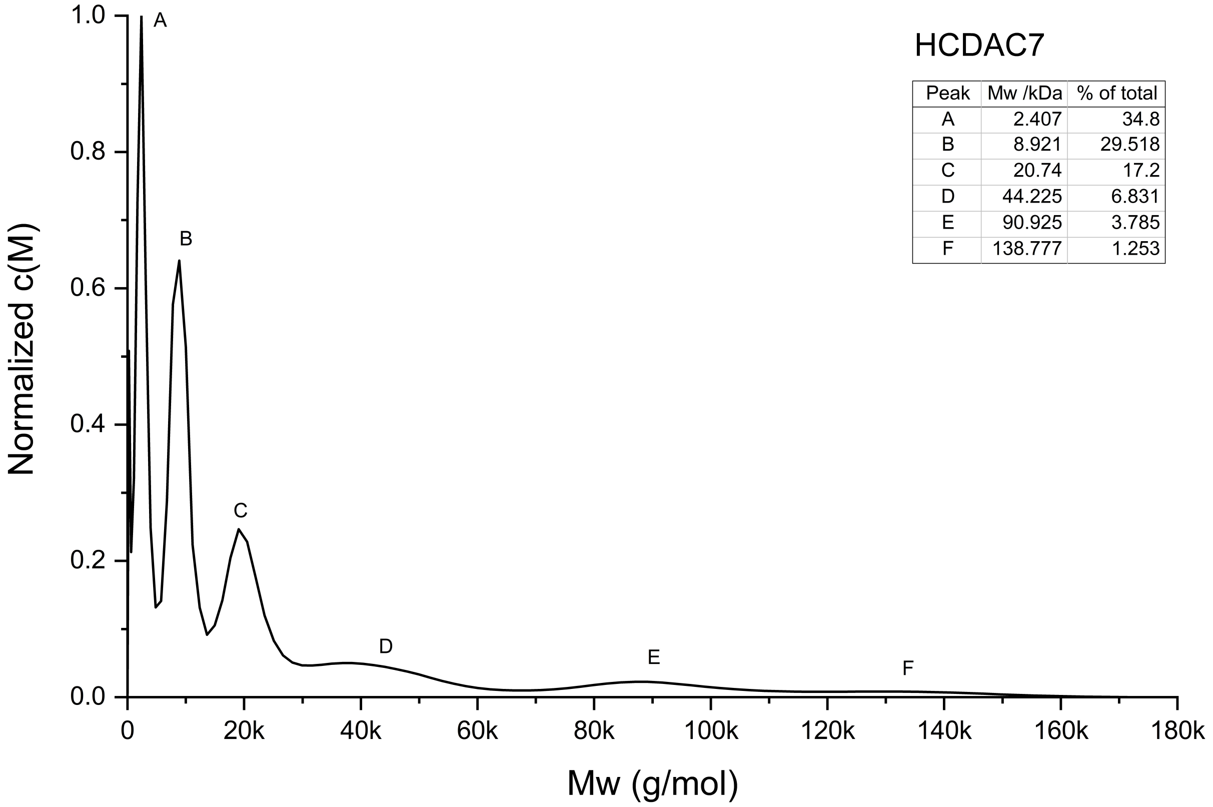
HCDAC7
Peak | Mw /kDa | % of total
A | 2.407 | 34.8
B | 8.921 | 29.518
C | 20.74 | 17.2
D | 44.225 | 6.831
E | 90.925 | 3.785
F | 138.777 | 1.253
Normalized c(M)
Mw (g/mol)
A
B
C
D
E
F
0.0
0.2
0.4
0.6
0.8
1.0
0
20k
40k
60k
80k
100k
120k
140k
160k
180k

(H)

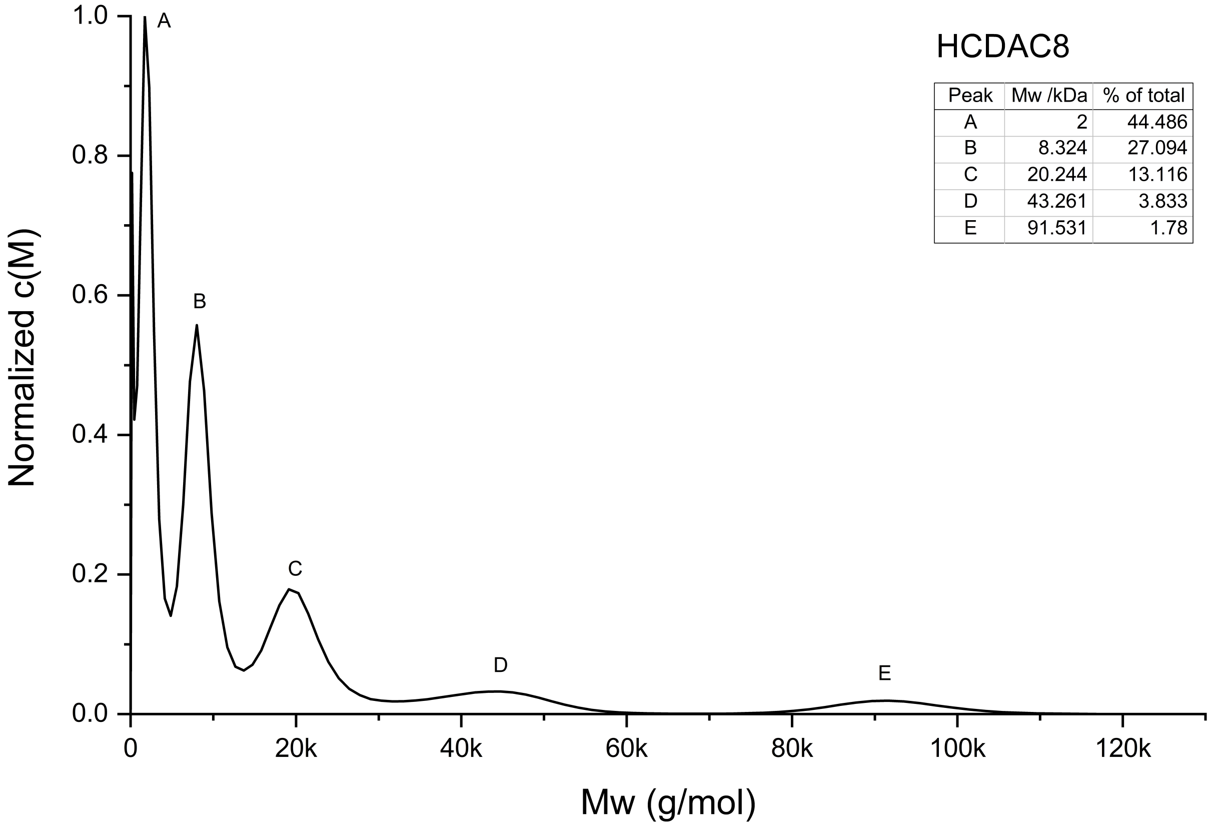
HCDAC8
Peak | Mw /kDa | % of total
A | 2 | 44.486
B | 8.324 | 27.094
C | 20.244 | 13.116
D | 43.261 | 3.833
E | 91.531 | 1.78
Normalized c(M)
Mw (g/mol)
A
B
C
D
E
0.0
0.2
0.4
0.6
0.8
1.0
0
20k
40k
60k
80k
100k
120k

5

(I)

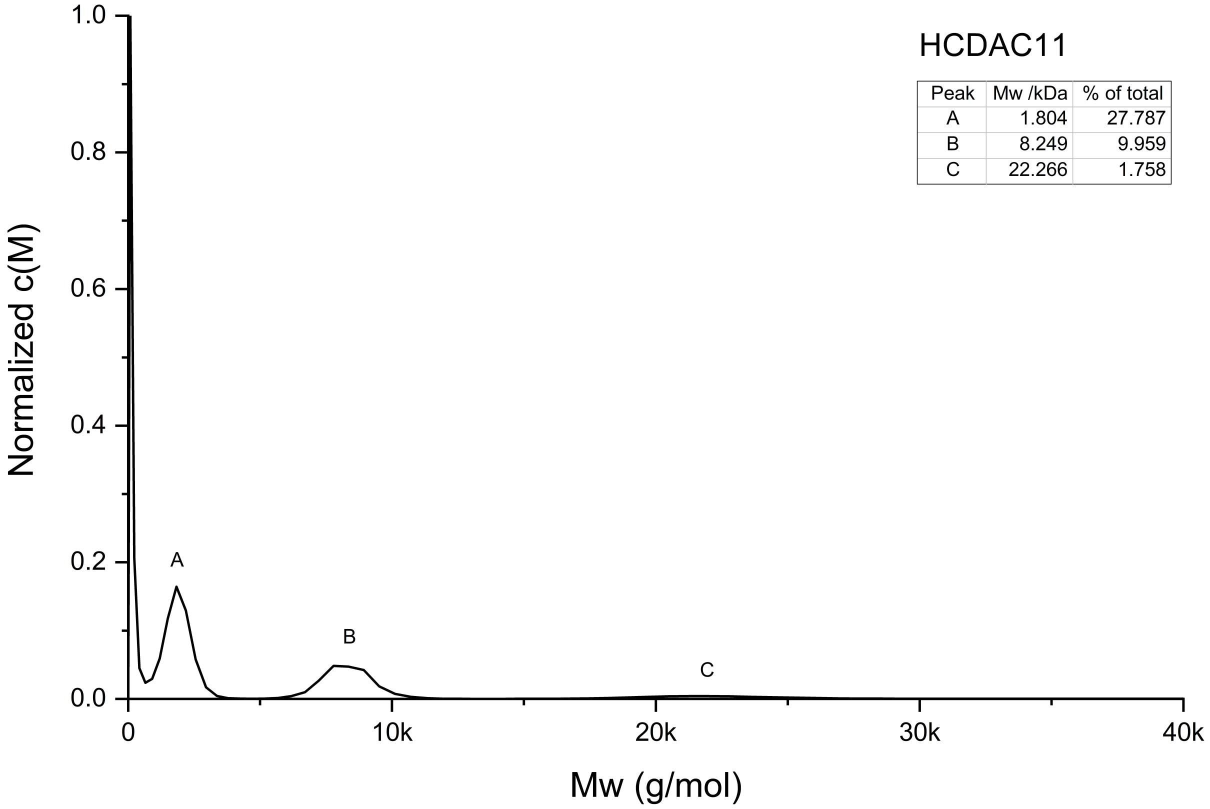

(J)

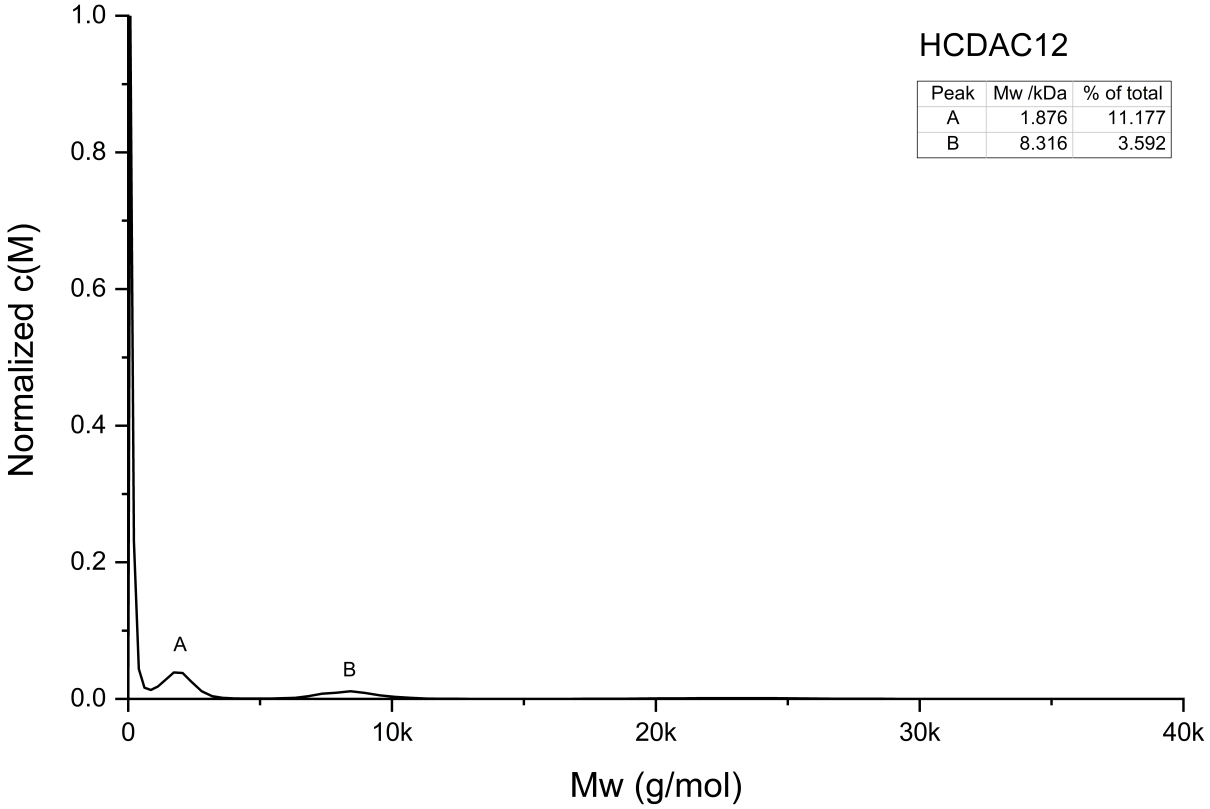

Figure S2. (1): Normalized calculated molar mass distributions c(M) for hydrophobically modified cationic cellulose polymers. The table in the plot shows the "PEAK INFO" of the different peaks in c(M) distribution using a high-resolution sedimentation coefficient distribution c(s) in SEDFIT

program. **(A)** for HCDAC1 with the determined density of 1.316 g/ml and a best-fit averaged friction ration of 1.116. **(B)** for HCDAC2 with the determined density of 1.214 g/ml and a best-fit averaged friction ration of 1.182. **(2): (continued from previous page): (C)** for HCDAC3 with the determined density of 1.328 g/ml and a best-fit averaged friction ration of 1.183. **(D)** for HCDAC4 with the determined density of 1.266 g/ml and a best-fit averaged friction ration of 1.153. **(3): (continued from previous page): (E)** for HCDAC5 with the determined density of 1.337 g/ml and a best-fit averaged friction ration of 1.150. **(F)** for HCDAC6 with the determined density of 1.309 g/ml and a best-fit averaged friction ration of 1.139. **(4): (continued from previous page): (G)** for HCDAC7 with the determined density of 1.329 g/ml and a best-fit averaged friction ration of 1.157. **(H)** for HCDAC8 with the determined density of 1.338 g/ml and a best-fit averaged friction ration of 1.186. **(5): (continued from previous page): (I)** for HCDAC11 with the determined density of 1.320 g/ml and a best-fit averaged friction ration of 1.187. **(J)** for HCDAC12 with the determined density of 1.336 g/ml and a best-fit averaged friction ration of 1.187.
